# Monocyte-endothelial cell interactions in vascular and tissue remodeling

**DOI:** 10.3389/fimmu.2023.1196033

**Published:** 2023-07-07

**Authors:** Mireia Medrano-Bosch, Blanca Simón-Codina, Wladimiro Jiménez, Elazer R. Edelman, Pedro Melgar-Lesmes

**Affiliations:** ^1^ Department of Biomedicine, School of Medicine, University of Barcelona, Barcelona, Spain; ^2^ Biochemistry and Molecular Genetics Service, Hospital Clínic Universitari, Instituto de Investigaciones Biomédicas August Pi i Sunyer (IDIBAPS), Centro de Investigación Biomédica en Red de Enfermedades Hepáticas y Digestivas (CIBERehd), Barcelona, Spain; ^3^ Institute for Medical Engineering and Science, Massachusetts Institute of Technology, Cambridge, MA, United States

**Keywords:** monocyte, macrophage, endothelial cell, angiogenesis, tumor, cardiovascular diseases, liver diseases.

## Abstract

Monocytes are circulating leukocytes of innate immunity derived from the bone marrow that interact with endothelial cells under physiological or pathophysiological conditions to orchestrate inflammation, angiogenesis, or tissue remodeling. Monocytes are attracted by chemokines and specific receptors to precise areas in vessels or tissues and transdifferentiate into macrophages with tissue damage or infection. Adherent monocytes and infiltrated monocyte-derived macrophages locally release a myriad of cytokines, vasoactive agents, matrix metalloproteinases, and growth factors to induce vascular and tissue remodeling or for propagation of inflammatory responses. Infiltrated macrophages cooperate with tissue-resident macrophages during all the phases of tissue injury, repair, and regeneration. Substances released by infiltrated and resident macrophages serve not only to coordinate vessel and tissue growth but cellular interactions as well by attracting more circulating monocytes (e.g. MCP-1) and stimulating nearby endothelial cells (e.g. TNF-α) to expose monocyte adhesion molecules. Prolonged tissue accumulation and activation of infiltrated monocytes may result in alterations in extracellular matrix turnover, tissue functions, and vascular leakage. In this review, we highlight the link between interactions of infiltrating monocytes and endothelial cells to regulate vascular and tissue remodeling with a special focus on how these interactions contribute to pathophysiological conditions such as cardiovascular and chronic liver diseases.

## 1 Introduction

Monocytes and monocyte-derived macrophages (MDM) are plastic cells from the innate immune system that exhibit essential and distinct roles in homeostasis, immune response, inflammation and tissue repair (1). They contribute to a wide spectrum of diseases and are therefore attractive therapeutic targets (2–4). Potential therapeutic interventions involving monocyte or MDM modulation require an in-depth understanding of the mechanisms that govern their ontogeny, tissue infiltration, activation, and phenotype adaptation to the microenvironment. Monocytes can transdifferentiate to macrophages under extreme circumstances but generally do not significantly contribute to the majority of tissue macrophage populations in physiological conditions or in some particular inflammatory disorders (1). Most tissue macrophages are derived from embryonic precursors, established before birth, and maintained by self-renewal in adults (5, 6). Monocytes comprise ~4% of mice and 10% of humans blood nucleated cells with substantial reservoirs in the spleen and lungs that can be rapidly recruited to damaged tissues (7, 8). Circulating monocytes display a characteristic short half-life of around 20 hours (6), which is prolonged when they do transdifferentiate into macrophages to assist in establishing tissue-resident mononuclear phagocyte population (9). Indeed, monocytes appear as short-lived plastic cells meant to protect against pathogens or to harmonize vascular and tissue remodeling upon recruitment driven by chemokines and monocyte adhesion molecules (10). Therefore, monocytes are dynamic cellular components that can complete the functions of tissue-resident mononuclear phagocytes on demand. The greatest number of tissue-resident macrophages are configured as hepatic Kupffer cells (KCs). KCs are the most abundant tissue macrophages in mammalians, representing the 80–90% of total tissue macrophages (11). In physiological conditions, there is only a small number of MDM in the hepatic portal space (12). Mouse MDM can be distinguished from KCs by their differential expression on cell surface markers such as CD11b, F4/80, Ly6C, and macrophage colony-stimulating factor 1 receptor (CSF1R) (13). Human MDM are typically identified as CD14^+^, CC-chemokine receptor 2 (CCR2)^+^ cells (13). The liver is the first line defense against foreign molecules in particular those ingested. It is a dynamic filter and the core of body metabolism with a unique capacity among organs to regenerate to a physiological size even after two-thirds of its mass has been removed. This effect appreciated even in Greek mythology is the hallmark of health and as with Tityus and Prometheus regeneration occurs with partial hepatectomy. Excessive injury or significant disease can hamper such repair. Here, we discuss how monocyte-endothelial cell interactions and MDM participate in the regulation of angiogenesis and regeneration in liver diseases.

Injured cells release specific signals identified as damage-associated molecular patterns (DAMPs) that activate the immune system similarly to pathogen-associated molecular patterns (PAMPs), small molecular motifs released from bacteria or viruses (14). These endogenous molecules (calcium-binding proteins, structural and extracellular matrix (ECM) proteins, etc.) exhibit a wide array of cellular functions in homeostasis and as injury signals in a complex integrated network checking and balancing tissue repair and further damage. Tissue-specific macrophage subpopulations sense these signals and trigger endothelial cells (ECs), monocytes, and other immune cells to contain injury and initiate an immune response. Infiltrated MDM generally display a pro-inflammatory phenotype (M1-like) involving secretion of cytokines such as interleukin 1 (IL-1) and tumor necrosis factor (TNF-α) for inflammation propagation, and IL-12 for T helper 1 (TH1) lymphocyte activation and induction of the adaptive immune response (15). M1-like macrophages also release reactive oxygen and nitrogen species aimed at elimination of possible biological aggressors. However, all these cocktails of cytokines and free radicals also produce substantial collateral tissue damage to the host during the reaction against the insult. To prevent harmful effects to the tissue, regulatory mechanisms activate and promote macrophage apoptosis or polarization to a M2-like anti-inflammatory and pro-regenerative phenotype that facilitates wound healing (16). Indeed, damaged epithelial cells release alarmins, which induce IL-4 and IL-13 secretion by T-helper lymphocytes and other immune cells. Both IL-4 and IL-13 are major regulators of macrophage polarization to an anti-inflammatory M2-like phenotype (15). M2-like macrophages release vascular or fibroproliferative growth factors such as vascular endothelial growth factors (VEGFs) or transforming growth factor (TGFβ1), respectively (17). VEGF-A, for example, stimulates angiogenesis and vascular leakage (18). TGFβ1 induces fibroblast differentiation to myofibroblasts or phenomena of epithelial or endothelial to mesenchymal transitions (19, 20) that promote the synthesis of ECM components or tissue inhibitors of metalloproteinases (TIMP). The balance of M1/M2 profiles on MDM is crucial to understand prognostic in chronic diseases and especially in the context of liver cirrhosis (21) or in atherosclerosis and cardiovascular disease (22). In cardiovascular diseases, M1-like macrophages characterize progression lesions while regressing plaques are enriched in M2 macrophages (22). However, macrophage heterogeneity in atherosclerotic plaques may have only a partial semblance to M1-like and M2-like macrophage phenotypes. Indeed, it is yet necessary to identify gene-expression profiles and transcriptional pathways that underlie the distinctiveness and diversity of MDM in cardiovascular diseases.

## 2 Monocyte-endothelial cell interactions: molecular pathways

Monocytes interact with ECs under physiological or pathophysiological conditions to orchestrate inflammation, angiogenesis, or tissue remodeling (23). Indeed, the migration of monocytes from the circulation to peripheral organs during an inflammatory response depends on their interaction with ECs. These interactions are orchestrated and controlled by chemoattractant and adhesion molecules that allow monocyte trafficking (24). The initial rolling of monocytes along the activated endothelium results in a firm adhesion that eventually culminates with their transmigration at inflammation sites where they transdifferentiate into macrophages or dendritic cells (23).

### 2.1 Monocyte-attracting chemokines

Circulating monocytes have been classified into different subsets based on the chemokine receptors they express and the presence of specific surface molecules (25). In humans, monocytes are classified according to the presence of CD14 and CD16 on their surface (26, 27). CD14^++^CD16^–^ are known as classical monocytes, which are the most abundant in the bloodstream. Alternatively, CD14^++^CD16^+^ are referred as intermediate monocytes and, CD14^++^CD16^++^, as non-classical patrolling monocytes. However, in mice, only two subsets have been identified (28). One of these populations corresponds to CD14^+^ CD62 ligand (CD62L)^+^ CC-chemokine receptor 2 (CCR2)^+^, which is known as LY6C^hi^ or inflammatory monocytes (23, 29). The second population is similar to CD16^+^CCR2^–^ monocytes in humans, and it is known as LY6C^low^ or patrolling monocytes, that express high levels of CX3C-chemokine receptor 1 (CX3CR1) and low levels of CCR2 and LY6C (24).

Recruitment of LY6C^hi^ monocytes from the bone marrow is mediated by the binding of CC-chemokine ligand 2 (CCL2) (30), also known as monocyte chemoattractant protein-1 (MCP-1), and CCL7 (or MCP-3) to CCR2 (31, 32). Most cells express CCL2 in response to pro-inflammatory cytokines in infections (33). Thus, after many infections, circulating levels of CCL2 increase in both the serum and inflamed tissues, where CCL2 binds to CCR2 that is expressed on certain cell types (23). CCL7 expression is also stimulated by infections and contributes to LY6C^hi^ monocyte recruitment (23). Both CCL2 and CCL7 have shown important roles on monocyte recruitment, although the mechanism of action is still unclear (31, 32). Conversely, recruitment and survival of LY6C^low^ monocytes is mediated by the binding of CX3C-chemokine ligand 1 (CX3CL1), also known as fractalkine (FKN), to CX3CR1 (34).

Monocytes also express other CC-chemokine receptors, such as CCR1 and CCR5 (35) that bind to various cytokines including CCL3 (also known as MIP1α) and CCL5 (also denominated RANTES) (29). Both receptors display specialized roles in monocyte recruitment. CCR1 mediates monocyte arrest in fluid shear stress generated by blood flow. CCR5 is involved in monocyte spreading. They both contribute to transendothelial chemotaxis towards CCL5 gradients (36). However, none of these receptors have shown redundancy in cell recruitment during inflammation, which has implications in the development and progression of many diseases, including atherosclerosis and rheumatoid arthritis (29, 37, 38). This may be a consequence of the wide spectrum of cells expressing these chemokine receptors (39), so determining the specific role they play in monocyte recruitment is complex (23).

Other chemokines have also been suggested to play a role on monocyte recruitment (**Table 1**). Some examples are CCR6 (42), CCR7 (43), CCR8 (44), and CXC-chemokine receptor 2 (CXCR2) (45). Circulating monocytes express low levels of CCR6 and do not respond to CCL20, which explains that CCR6 does not display a significant role on the extravasation of monocytes from the circulation to the tissues, but it does on the migration or function of monocytes in inflammation (46, 47).

**Table 1:** Chemokines and chemokine receptors involving monocytes and endothelial cells.

Receptor	Chemokine ligands	Receptor expressed on	Ligands mostly expressed/secreted by	Reference
**CCR1**	CCL3 (MIP-1α), CCL5 (RANTES), CCL7 (MCP-3), CCL14 (HCC1)	Monocytes, T cells, dendritic cells	Monocytes macrophages, endothelial cells, T cells, dendritic cells, neutrophils, epithelial cells, fibroblasts	(40, 41)
**CCR2**	CCL2 (MCP-1), CCL8 (MCP-2), CCL7 (MCP-3), CCL13 (MCP-4), CCL16 (HCC4)	Monocytes, endothelial cells, dendritic cells	Monocytes, macrophages, endothelial cells, T cells, epithelial cells, fibroblasts	(40, 41)
**CCR5**	CCL3 (MIP-1α), CCL4 (MIP-1β), CCL5 (RANTES), CCL11 (eotaxin), CCL14 (HCC1), CCL16 (HCC4)	Monocytes, macrophages, endothelial cells, T cells, dendritic cells	Monocytes, macrophages, endothelial cells, T cells, epithelial cells, leukocytes	(40, 41)
**CCR8**	CCL1 (I309)	Monocytes, T cells, dendritic cells	Monocytes, T cells, mast cells	(40, 41)
**CXCR1**	CXCL8 (IL-8), CXCL6 (GCP2)	Monocytes, neutrophils	Macrophages, endothelial cells, T cells, epithelial cells, fibroblasts	(40, 41)
**CXCR2**	CXCL8 (IL-8), CXCL1 (GROα), CXCL2 (GROβ), CXCL3 (GROγ), CXCL5 (ENA-78), CXCL6 (GcP2)	Monocytes, microvascular endothelial cells, neutrophils	Monocytes, macrophages, endothelial cells, T cells, epithelial cells, fibroblasts, mast cells	(40, 41)
**CXCR3-B**	CXCL4 (PF4), CXCL9 (MIG), CXCL10 (IP-10), CXCL11 (I-TAC)	Microvascular endothelial cells, T cells, natural killer cells	Monocytes, endothelial cells, T cells, neutrophils, epithelial cells, fibroblasts, cancer cells	(40, 41)
**CX3CR1**	CX3CL1 (fractalkine)	Monocytes, macrophages, T cells, dendritic cells, smooth-muscle cells, natural killer cells	Endothelial cells, T cells	(40, 41)

### 2.2 Monocyte-attracting adhesion molecules

Monocyte migration to inflammatory sites is a multistep process involving many molecules (**Table 2**). The initial tethering and rolling of monocytes along the inflamed endothelium are mediated by selectins. Selectins are cell-surface proteins that interact with glycoprotein ligands to allow monocytes to bind weakly and reversibly to cytokine-activated ECs (24, 62). The selectins involved in this process are L-selectin (CD62L), P-selectin (CD62P) and E-selectin (CD62E). L-selectin, expressed on circulating monocytes, interacts with specific fucosylated sialoglycoproteins expressed on lymph node venules and inflamed or injured vascular endothelium (48, 49, 63). P-selectin glycoprotein ligand-1 (PSGL-1), P-selectin glycoprotein ligand-1 (PSGL-1), expressed on monocytes, interacts with P-selectinand E-selectin expressed on inflamed endothelium (50). Then, PSGL-1 can also interact with circulating monocytes expressing L-selectin and amplify the monocyte recruitment (52). This initial adhesion mediated by selectins reduces the rolling velocity of monocytes and allows the cells to interact with chemokines that are bound to inflamed ECs (64). ECs are activated by inflammatory cytokines such as TNF-α or IL-1β. This activation induces the expression of adhesion molecules such as E- and P-selectin, intercellular adhesion molecule 1 (ICAM1/CD54) and vascular cell-adhesion molecule 1 (VCAM1/CD106) that participate in monocyte migration (65).

**Table 2:** Monocyte adhesion molecules involved in transendothelial migration.

Adhesion molecule	Ligands	Ligand expressed on	Function	Reference
**L-selectin (CD62L)**	Fucosylated sialoglycoproteins	Endothelial cell	Rolling	(48, 49)
**PSGL-1**	P-selectin (CD62P) E-selectin (CD62E)	Endothelial cell	Rolling	(50, 51)
**PSGL-1**	L-selectin	Monocyte	Monocyte recruitment	(52, 53)
**LFA1** **(αLβ2-integrin or CD11a–CD18)**	ICAM1/CD54	Endothelial cell	Arrest	(24, 54, 55)
**VLA4** **(α4β1-integrin or CD49d–CD29**	VCAM1/CD106	Endothelial cell	Arrest	(24, 54, 55)
**Mac1** **(αMβ2-integrin or CD11b–CD18**	ICAM1/CD54 among others	Endothelial cell	Arrest	(56)
**LFA1** **(αLβ2-integrin or CD11a–CD18)**	JAM-A	Endothelial cell	Transmigration	(57)
**VLA4** **(α4β1-integrin or CD49d–CD29**	JAM-B	Endothelial cell	Transmigration	(58)
**Mac1** **(αMβ2-integrin or CD11b–CD18**	JAM-C	Endothelial cell	Transmigration	(59)
**αXβ2-integrin (CD11c–CD18)**	JAM-C	Endothelial cell	Transmigration	(59)
**PECAM1** **(CD31)**	PECAM1	Endothelial cell	Transmigration	(60)
**CD99**	CD99	Endothelial cell	Transmigration	(61)

Chemokines can bind to transmembrane heparan sulphate proteoglycans on the luminal surface of vascular ECs to be presented to monocytes (66). The main proteoglycans identified as chemokine-binders include CD44, syndecan 1 and syndecan 4 (which bind CCL5) and syndecan 2 (which interacts with CXCL8) (66). Chemokines bind to G-protein-coupled receptors (GPCRs) of monocytes and induce inside-out signals that result in integrin activation. GPCRs activate specific Gi and Gq heterotrimeric proteins and their downstream effectors. Two key guanosine triphosphatases (GTPases), RhoA and Rap1, have been implicated in chemokine activation of integrins (67, 68). Rap1 is a small GTPase of the RAS family that cycles between an inactive GDP-bound form and an active GTP-bound form (54). Activated Rap1 binds RAPL, and the complex activates the integrin by binding to the alpha-chain of integrin (69). Rap1 also binds to Rap-interacting adapter molecule (RIAM), which recruits talin (70). Talin is a cytoskeletal protein that binds to the beta-chain of the integrins, thereby triggering integrin activation (71, 72). Integrin activation induces conformational changes initiated at the alpha and beta subunit cytoplasmic tails and transmitted to their extracellular domain (73). This inside-out signaling activates integrins such as lymphocyte function-associated antigen 1 (LFA1; also known as αLβ2-integrin and CD11a–CD18) and very late antigen 4 (VLA4; also known as α4β1-integrin, and CD49d–CD29). Activated β2 and α4-integrins ensure the arrest of monocytes and the formation of firm adhesions by binding ICAM1 and VCAM1 respectively, which are expressed by inflamed endothelial cells (54) [**Table 2**]. Other integrins such as the macrophage receptor 1 (Mac1; also known as αMβ2-integrin and CD11b–CD18) and αXβ2-integrin are also activated *via* this signaling pathway (56).

### 2.3 Monocyte transmigration

**Figure 1:**
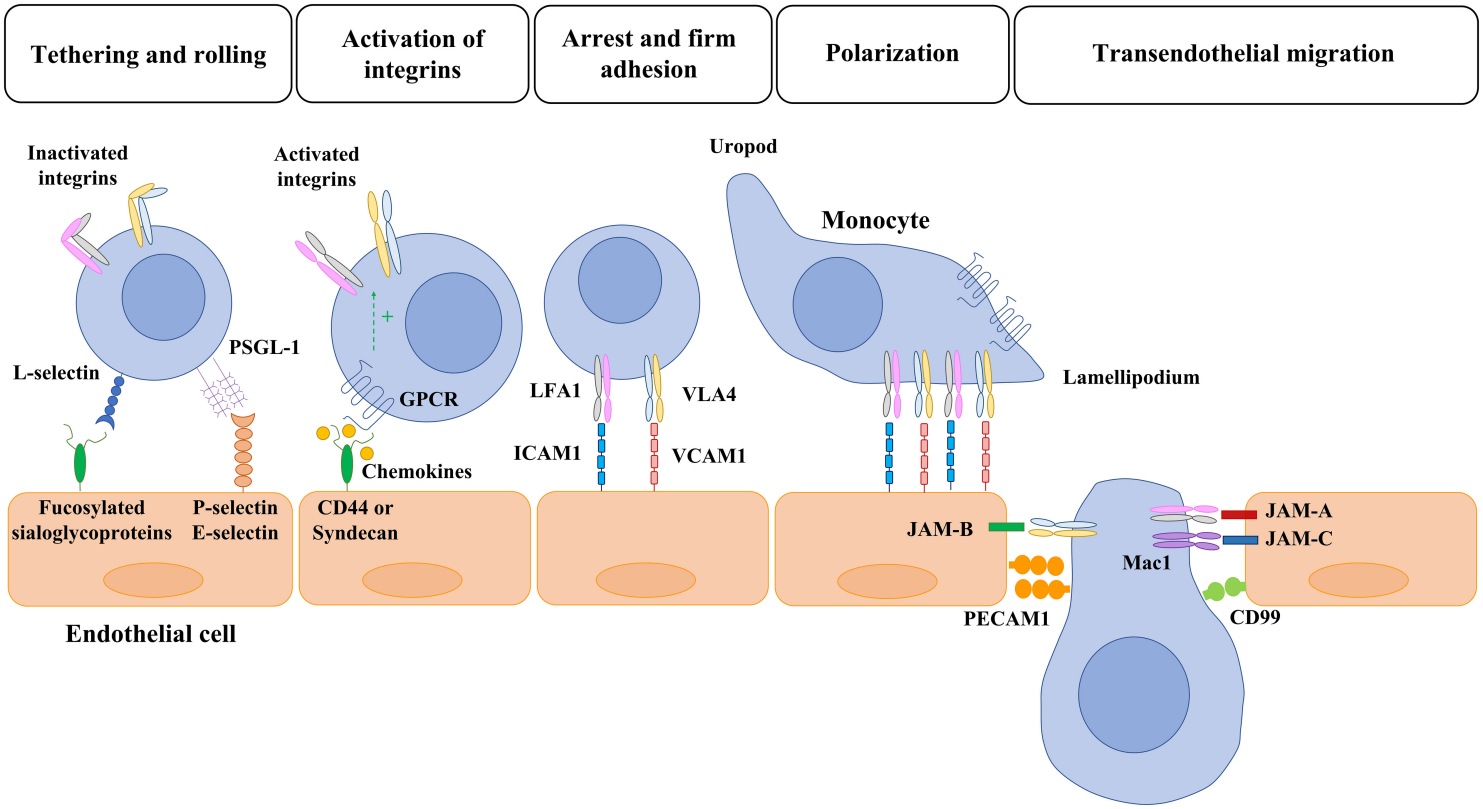
Overview of monocyte-endothelial cell interaction and transmigration. Monocyte migration to inflammatory sites is a multistep process with many molecules involved. First, selectins mediate the initial tethering and rolling of monocytes along the cytokine-activated endothelial cells. Then, the monocyte interacts with chemokines that are bound to transmembrane heparan sulphate proteoglycans (CD44 and sydecan) expressed in the endothelium. The activation of GPCR leads to the activation of integrins and the consequent monocyte arrest mediated by the interaction of LFA1 and VLA4 with ICAM1 and VCAM, respectively. Once a monocyte establishes firm adhesion to the vascular endothelium, it undergoes a morphological change known as polarization, in which chemokine receptors and activated integrins redistribute to the leading edge. After polarization, monocytes migrate toward interendothelial junctions and then transmigrate into the underlying tissues. The members of the JAM family expressed by endothelial cells (JAM-A, JAM-B, JAM-C) interact with the activated integrins of monocytes (LFA1, VLA4, Mac1) and allow the transmigration through tight junctions. Lastly, PECAM-1 (CD31) and CD99 hemophilic engagement and endothelial retraction lead to monocyte extravasation. PSGL: P-selectin glycoprotein ligand-1; GPCR: G-protein-coupled receptors; LFA1: lymphocyte function-associated antigen 1; VLA4: very late antigen 4; ICAM1: intercellular adhesion molecule 1 (ICAM1/CD54); VCAM: vascular cell-adhesion molecule 1; Mac1: macrophage receptor 1; PECAM: platelet/endothelial cell-adhesion molecule 1.

The firm adhesion of a monocyte to the vascular endothelium results in a morphological and phenotypical change known as polarization (**Figure 1**) (74). Polarization involves the formation of two different regions: the lamellipodia at the leading edge and the uropod at the tail of the monocyte (74). Polarization involves reorganization of the cytoskeletal proteins, intracellular regulatory molecules, chemoattractant receptors and integrins (75). F-actin changes from radially symmetric around the cell to accumulated in the leading edge (76). Chemokine receptors redistribute to the leading edge, while other adhesion molecules, such as CD44, accumulate at the uropod (75). In addition, high affinity integrins mobilize to the leading edge and low affinity integrins to the uropod (54). Attachment of integrins at the leading edge and detachment at the uropod occurs during migration (77). Several signaling pathways contribute to polarization including Rho family GTPases, GTPase Rap1, protein kinases, and lipid kinases (75). GTPase Rap1 has also been described as a key molecule in integrin activation and redistribution during leukocyte polarization (54). RAP1 and RAPL control the polarized recruitment of integrin clusters to the lamellipodium (69). RHOA, another member of the RAS superfamily, also participates in integrin clustering by activating RHO-associated coiled-coil containing protein kinase 1 (ROCK1), which phosphorylates the actin cytoskeleton (24). GPCR downstream signals also activate PI3K, which participates in monocyte polarization *via* activation of the atypical protein kinase C-ζ (PKC-ζ) and formation of the polarity complex (consisting of partitioning defective 6 (PAR6)/PKC-ζ/lethal giant larvae, LGL). PKC-ζ signaling facilitates integrin lateral mobility (after integrin is in its high affinity form) due to the mobilization of new lipid membrane to the leading edge (78). The polarized recruitment of integrins clusters to the lamellipodium results in polarized adhesion and then migration (24). After polarization, monocytes migrate to the interendothelial junctions.

Adjacent ECs are connected by a wide array of endothelial junctions: tight junctions, adherens junctions and gap junctions (79). Tight and adherens junctional transmembrane proteins mediate cell adhesion by homophilic interactions and form a zipper-like structure along the cell border. This adhesion is reorganized during monocyte transendothelial migration (79). Indeed, tight and adherens junctional proteins play a critical role in this process (**Table 2**) (24). Tight junctions are localized at the apical site of the interendothelial junctions and form a close contact between adjacent ECs. Tight junctions are composed of occludins, claudins and junctional adhesion molecules (JAMs), but only JAMs have been described to participate directly in the transendothelial migration of monocytes (80). Three members of the JAM family have been described: JAM-A, JAM-B, and JAM-C. JAMs from ECs bind to monocyte integrins. LFA1 has been identified as a ligand for JAM-A (57). JAM-B has been described to bind to α4β1-integrin and JAM-C to αMβ2-integrin (also known as MAC1 and CD11b– CD18) and αXβ2-integrin (also known as CD11c–CD18) (58, 59). Moreover, JAM-C is specifically required to prevent reverse transmigration of monocytes back to the vascular lumen (81). The interaction between the members of the JAM family and the integrins expressed by monocytes allows the transmigration through tight junctions (**Figure 1**). Then, monocytes transmigrate through the adherens junctions. The main component of adherens junctions is the vascular endothelial cadherin (VE-Cadherin), an endothelial-specific protein anchored to the cytoskeleton and responsible for endothelial tightness against leakage (24). VE-cadherin plays an important role on the control of vascular permeability and integrity but does not interact with monocyte proteins to facilitate transmigration. In contrast, molecules present in adherens junctions such as platelet/endothelial cell-adhesion molecule 1 (PECAM1) and CD99 participate in this process by homophilic interactions (24, 60, 61) (**Figure 1**). VE-cadherin is crucial to regulate endothelial permeability because it participates in adherens junctions dismantling of and EC retraction, which is required to complete the transmigration of monocytes (60). The clustering of selectins and/or VCAM/ICAM induces an activation of RhoA and an increase in intracellular free calcium within the endothelial cells (82). This results in the activation of the calmodulin-dependent enzyme myosin light chain kinase (MLCK), thereby causing a conformational change in myosin II (83) and the phosphorylation of the VE-cadherin cytoplasmic tail (82). ICAM-1 engagement also results in the activation of Src and PYK2 kinases, which also phosphorylate VE-Cadherin (84). The phosphorylation on Tyr658 and Tyr731 induce the dissociation of VE-cadherin from the cytoskeleton and allow the splitting of EC junctions (82). These changes lead to the increase in vascular permeability and allow monocyte extravasation (60). Monocytes mostly transmigrate through junctions between adjacent ECs (paracellular transmigration) although monocytes can also migrate through ECs (transcellular transmigration) by the formation of vesiculo-vacuolar organelles (85).

The reorganization of interendothelial junctions during inflammation is temporally and spatially regulated by inflammatory mediators and leukocyte transendothelial migration (86). These changes are reversible, and endothelium quiescence and vascular permeability are restored once the triggering cause is removed. However, in some pathological conditions such as chronic inflammation and atherosclerosis, the ECs remain activated and the interendothelial junctions become instable (87). This instability causes an impairment in the endothelial barrier function leading to uncontrolled leukocyte migration and vascular leakage. Defects in the organization of endothelial cell junctions can lead to vascular malformations, vascular fragility and rupture, and appearance of hemorrhages and edema (88). Loss of barrier integrity is a common feature in several vascular disorders including anaphylaxis, diabetic microangiopathy, angioedema, or cancer and metastasis (85). Endothelial junctions not only mediate adhesion but also trigger intracellular signals that communicate cell position, limit growth and apoptosis. They are essential to maintain vascular integrity and homeostasis (88). Furthermore, the reorganization of interendothelial junctions and loosening of cell-cell adhesion is also required for other physiological processes such as angiogenesis (85).

## 3 Monocyte-endothelial cell interactions in angiogenesis and vasculogenesis

Angiogenesis is the process of formation of new endothelium-lined channels from pre-existing blood vessels (89). Vasculogenesis refers to the creation of new blood vessels, mainly in the embryo, involving differentiation of angioblasts or endothelial progenitor cells (90). In adults, blood vessel formation may be defined by either angiogenesis or arteriogenesis depending on the initial trigger and the final vessel structure. Whereas angiogenesis is usually induced by hypoxia, arteriogenesis is induced by physical forces and, among them, primarily by increased fluid shear stress. Arteriogenesis entails the remodeling of pre-existing arterio-arteriolar anastomoses *via* recruitment of smooth muscle cells to fully established and functional arteries (89). In contrast, angiogenesis (capillary sprouting) results in higher capillary density but without the presence of vascular smooth muscle cells. Increasing evidence implicates blood monocytes in the selection of vascular sprouting points and assistance in vascular bridging (90, 91). Indeed, monocytes and MDM release a combination of chemokines, growth factors, and proteases that may simultaneously participate in immune cell attraction, basement membrane degradation, and endothelial proliferation (92, 93).

### 3.1 Monocyte-endothelial cell interactions in angiogenesis

**Figure 2:**
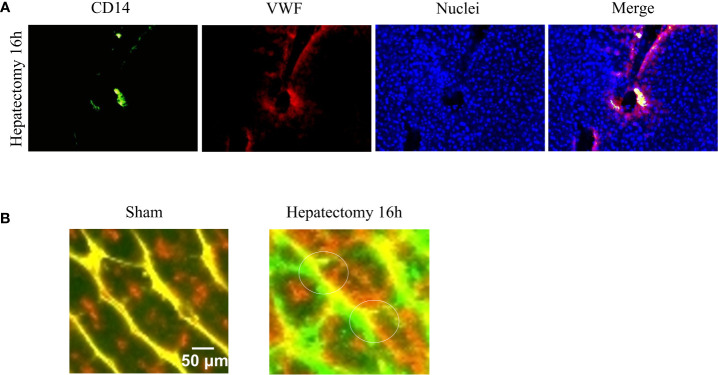
Monocyte-endothelial cell interactions and vascular sprouting occurring after hepatectomy. **(A)** Images of vessels from liver sections identified by staining for von Willebrand factor (VWF, in red) and recruited monocytes by staining for CD14 (in green). Nuclei were stained by DAPI (in blue). Initial contacts of recruited monocytes (in yellow) take place in portal areas. **(B)** Amplification of multiphoton images (vessels in green and yellow, macrophages in red), visualized vascular buds (white circles) surrounded by spread recruited macrophages 16 hours after hepatectomy. Images reprinted with permission from “Melgar-Lesmes, P.; et al. Monocyte-endothelial cell interactions in the regulation of vascular sprouting and liver regeneration in mouse. J Hepatol. 2015; 63 4):917-25. doi: 10.1016/j.jhep.2015.05.011”.

Vascular sprouting or angiogenesis is one, but not the only, process of blood vessel formation in the adult. Ordered angiogenesis has long been considered a critical mechanism for optimal wound healing. Little is known about the molecular basis by which leading ECs at vascular sprouts (endothelial ‘‘tip’’ cells) are designated to grow and elongate to form new blood vessels. Some investigations show that direct interactions of monocytes with ECs may be a driving force to stimulate endothelial proliferation (93, 94) and to mediate the fusion of endothelial tip cells (95). Indeed, some investigators have demonstrated that circulating monocytes are selectively recruited to certain regions of regenerating livers after hepatectomy (**Figure 2A**), particularly surrounding sprouting points (91) (**Figure 2B**).

Monocyte recruitment initiates in portal areas and expands to the rest of hepatic tissue in correlation with vasodilation and the expression of the inducible form of nitric oxide synthase (iNOS). iNOS is upregulated in injury and induces the synthesis of the vasodilator and proangiogenic substance NO (96). Indeed, angiogenesis initiates with vasodilation, a process involving NO (89). Then, vascular permeability increases in response to proangiogenic factors such as VEGF, plasma proteins extravasate and pave the ground with a provisional path for the migration of ECs. In this scenario, vascular permeability is affected by the formation of fenestrations, vesiculo–vacuolar organelles and the relocation of PECAM-1 and VE–cadherin, which implicates Src kinases (97, 98). Although angiogenesis requires increased permeability, vascular leakage, and release of permeability factors such as VEGF, it needs to be finely regulated to avoid water and sodium imbalance, circulatory collapse, intracranial hypertension, or ascites (18) among other pathological conditions. Either VEGF or other proangiogenic factors such as angiopoietins may be released by monocytes and MDM to regulate vascular permeability (92, 99). Angiopoietin 1 (Ang-1), a ligand of the endothelial Tie2 receptor, is an endogenous inhibitor of vascular permeability *via* strengthening of endothelial junctions (100). In contrast, angiopoietin 2 (Ang-2) is a partial agonist/antagonist of Tie2, thus promoting vascular leakage (101, 102). Indeed, Ang-2 is involved in the processes of detachment of smooth muscle cells and loosening of the ECM (103). In addition, MDM release different matrix metalloproteinases (MMP) that may activate or liberate a myriad of growth factors (bFGF, VEGF, IGF-1, etc) retained within the extracellular matrix (104, 105).

**Figure 3:**
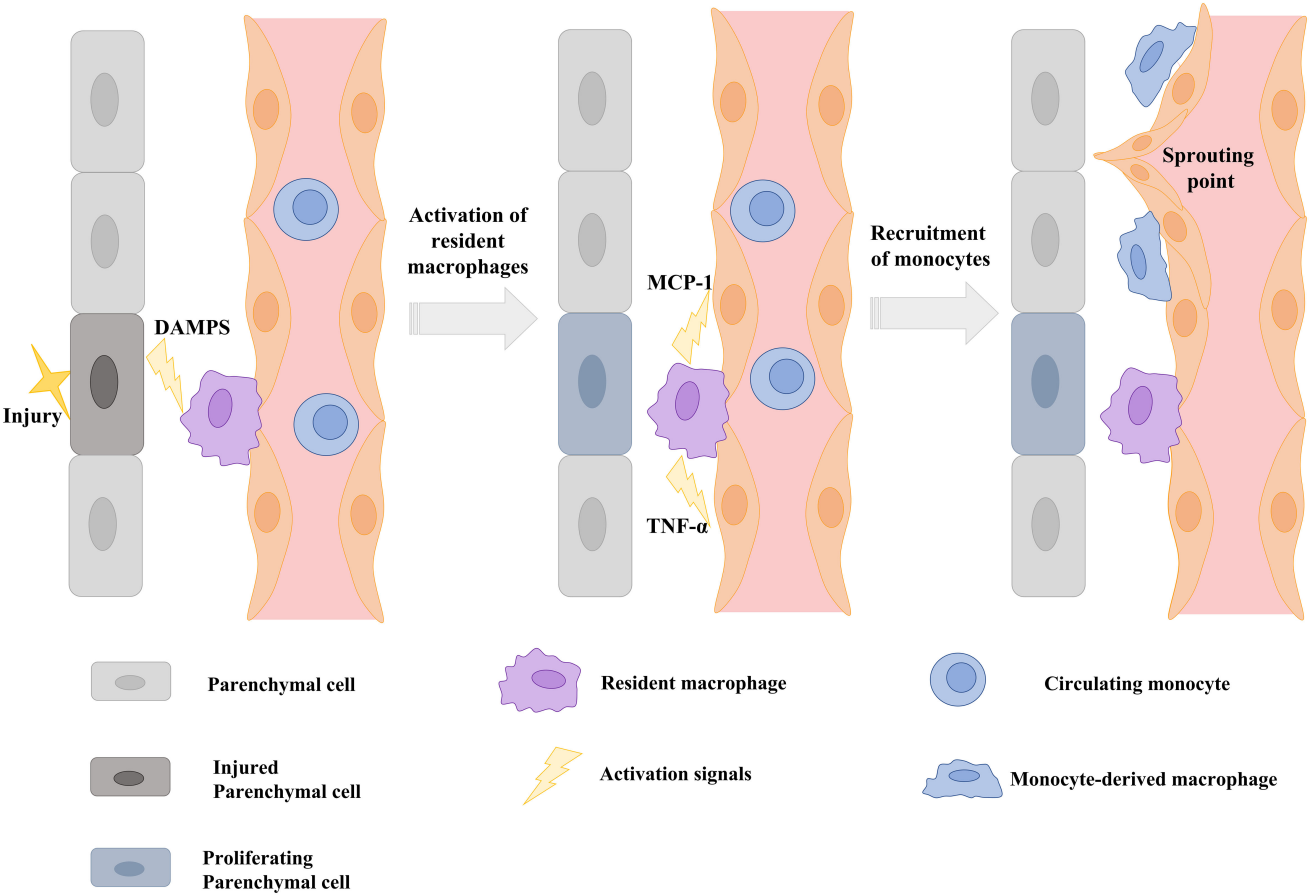
Schematic illustration showing that signals from injured parenchymal cells stimulate the release of cytokines and chemokines from resident macrophages and the induction of monocyte adhesion molecules on the endothelium to stimulate vascular sprouting. DAMPS: Danger-associated molecular patterns; TNF-α: tumor necrosis factor alpha; MCP-1: Monocyte chemoattractant protein-1. Scheme modified and adapted from “Melgar-Lesmes, P.; et al. Monocyte-endothelial cell interactions in the regulation of vascular sprouting and liver regeneration in mouse. J Hepatol. 2015 Oct;63 (4):917-25. doi: 10.1016/j.jhep.2015.05.011”.

Substances released by parenchymal cells (damage-associated molecular patterns) and resident macrophages (e.g. TNF-α) during tissue injury serve not only to coordinate vessel and tissue growth, but also cellular interactions, attracting circulating monocytes (e.g. MCP-1) and inducing neighboring ECs to expose monocyte adhesion molecules such as those described in the section 2.2 of this review (106) (**Figure 3**).

**Figure 4:**
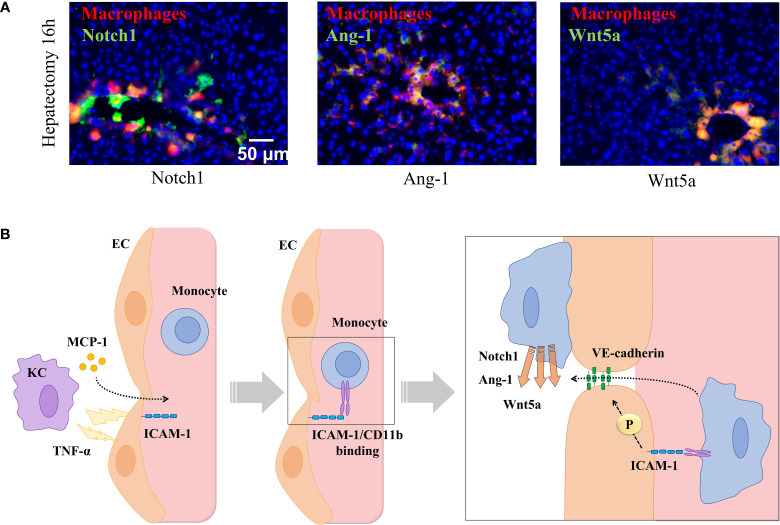
Monocyte-endothelial cell interactions locally release sprouting factors. **(A)** Hepatic staining of recruited monocytes (CD14, in red) enhancing the local expression of sprouting-related factors Wnt5a, Notch1, and Ang-1 (in green) in contact points of vessels (in yellow) in portal areas 16 hours post-hepatectomy. **(B)** Signals released by resident macrophages (KCs) recruit monocytes to selected areas driving the sprouting and angiogenic process. KCs deliver MCP-1 to blood stream and TNF-α towards nearby ECs to promote the expression of ICAM-1, which recruit monocytes and allow phosphorylation of the interendothelial VE-cadherin allowing monocyte migration throughout the vessel where they locally deliver sprouting factors (Notch1, Ang-1, and Wnt5a). Images reprinted with permission from “Melgar-Lesmes, P.; et al. Monocyte-endothelial cell interactions in the regulation of vascular sprouting and liver regeneration in mouse. J Hepatol. 2015;63 (4):917-25. doi: 10.1016/j.jhep.2015.05.011”.

Circulating monocytes detect attracting signals and interact with ECs to initiate endothelial disruption and monocyte translocation to local sprouting points. It has been demonstrated that the number of interactions of recruited monocytes with liver vascular network during regeneration is directly associated with phosphorylation and disruption of VE-cadherin connections (91). This uncoupling of inter-EC connectivity mediated by VE-cadherin is critical to the plasticity of the selected endothelial tip cell and for EC migration and elongation (107). MDM also serve as chaperones of endothelial sprouting by locally secreting proliferative factors, such as Wnt5a and Ang-1 and the stalk cell stabilizer Notch1 (**Figure 4**).

The release of Wnts from infiltrating monocytes and, namely, the contribution of Wnt5a and non-canonical ligands is especially important to harmonize angiogenesis in inflamed vessels (108).. Recruited monocytes are important beyond the priming phase of angiogenesis and throughout the process of tissue regeneration. Indeed, leukocyte adhesion molecules such as P-selectin, CCR2, and VCAM-1 upregulate during tissue regeneration and maintain the number of rolling interactions and the likelihood of recruiting monocytes. Specifically, the adhesion molecule ICAM-1 is upregulated after hepatectomy, but its gene expression is significantly downregulated when monocyte interactions and VE-cadherin phosphorylation are maxima. This points to a precise regulation of this adhesion molecule by monocyte interactions in accordance with the requirements of endothelial sprouting and the subsequent hepatic mass expansion. In fact, ICAM-1 activation is an important signaling pathway to trigger vascular sprouting (109, 110) and arteriogenesis (111). ICAM-1 is then an excellent candidate to understand the role of direct interactions of monocytes with endothelium to regulate vascular and tissue regeneration. Indeed, gene suppression of any of the subunits of the monocyte receptor Mac-1 (CD11b/CD18) disrupts wound healing in mice (112, 113). Moreover, suppression of the CD11b gene drops survival in mice undergoing partial hepatectomy and hinders vascular and liver mass regeneration in line with a reduced infiltration of circulating monocytes into the hepatic vascular network (91). Interestingly, the lack of CD11b also induces TNF-α expression, which can also directly promote VE-cadherin phosphorylation to replace the missing effects of monocyte-endothelial cell interactions (114). However, this increase in TNF- α is followed by an intense vascular leakage and a disorganized and aberrant vascular network after hepatectomy (91). Indeed, exacerbated increase of TNF-α by monocytes or MDM is associated to suppressive effects on wound healing in mice (115). To regulate these vascular disorders, tissue macrophages, such as KC, may progressively move into vessel walls from their static state and location in an attempt to replace the role of monocyte-EC interactions in the control of vascular and tissue growth in ICAM-1 KO mice (91). Thus, macrophage interaction with ICAM-1 in endothelium seems a critical step in the regulation of the endothelium integrity and in the control of TNF-α expression during angiogenesis after hepatectomy.

### 3.2 Monocyte-endothelial cell interactions in vasculogenesis

The molecular basis of vasculogenesis (in the embryo and from endothelial progenitors) diverges from that of pathological angiogenesis in the adult. The formation of the vascular network in the embryonic phase requires several sequential steps combining both mechanisms, vasculogenesis and angiogenesis. Vasculogenesis gives rise to the heart and the initial embryonic vascular plexus and surrounding membranes comprising the yolk sac circulation (116). Angiogenesis accounts for the expansion and remodeling of this vascular tree *via* endothelial sprouting and intussusceptive microvascular growth. However, little is known of any possible direct implication of monocyte or macrophage-EC interactions to drive vasculogenesis. The fact is that the macrophage is one of the first blood cell lineages to originate during embryonic development. Macrophages generate and grow surrounded by ECs and vasculogenesis occurs in parallel with hematopoiesis (116). Studies in mice have revealed that embryonic macrophages arise during three different waves of hematopoiesis: primitive hematopoiesis, erythro-myeloid progenitor (EMP) generation, and definitive hematopoietic stem cell-mediated hematopoiesis (116). Macrophages are then distributed through most developing organs (117). These initial macrophages derived during primitive hematopoiesis behave as a mediator between neovascularization and organ architecture during fetal organogenesis (118). Further recent findings using inducible Csf1r promoter-driven-Cre ROSAYFP reporter mice support a direct relationship between blood and endothelial lineages (119). These authors found that circulating EMP contribute to ECs in vascular network in multiple tissues (liver, brain, heart, lung, and yolk sac), and their interaction continues throughout adulthood (119). Other authors have found evidence on the active role of macrophages to promote vasculogenesis during retinal neovascularization (120). They have shown that macrophages boost the recruitment and differentiation of bone marrow-derived cells (BMCs). Mechanistically, they found that specifically M2-like macrophages affected the migration and activation of BMCs *via* secretion of VEGF and stromal cell-derived factor-1 (SDF-1). This is consistent with another investigation that demonstrated a higher recruitment of BMCs by SDF-1 and hepatocyte growth factor (HGF), released by the interaction between macrophages and matrix-embedded endothelial cells (MEECs) during liver regeneration (121). Namely, HGF stimulated the expression of the receptors CXCR4 and CXCR7 (122), that promoted the mobilization of endothelial progenitors to the blood stream and their recruitment into injured liver. In turn, incorporated endothelial progenitors secreted more HGF promoting positive feedback and the formation of new blood vessels that irrigated the implants and the ischemic tissue (121).

Vascular development is one of the earliest organogenesis events in embryonic development. A primitive vascular tree provides a basic path for circulating cells and guarantees the supply of nutrients. EC differentiation arises during gastrulation when cells invaginate to form the mesoderm. This process occurs around the embryonic day E7.25 in different clusters of cells in the extra-embryonic yolk sac, called blood islands (123). Blood islands are disposed of primitive hematopoietic cells in the center and aligned endothelial cells in the periphery (124). Primitive hematopoiesis produces unipotent myeloid progenitors that may uniquely generate the macrophage lineage (124), bipotent progenitors of erythrocytes, and megakaryocytes (125). Then, these progenitors are mobilized to the blood circulation around E8.0 (124). The first embryonic-derived macrophages are detected in the yolk sac at E9.0 (126). Yolk sac-derived macrophages colonize first the developing brain by E9.5 and then the rest of the embryonic tissues by E12.5 (123, 124). Although primitive macrophages are generated directly from progenitors without going through a monocyte intermediate (126), it remains elusive the possible interplay with endothelium during this phase to understand positioning and growth of the fetal vascular tree during organogenesis. Both vasculogenesis and macrophage generation occur in parallel in space and time, but how direct interactions of nascent macrophages or monocytes with ECs contribute to vasculogenesis need further investigation.

### 3.3 Monocyte-endothelial cell interactions in tumor angiogenesis

Infiltration of circulating monocytes to the tumor microenvironment (TME) is critical for tumor angiogenesis (127). It is well-known that inflammation is a starting event to attract and recruit monocytes and a driving force for monocyte-endothelial cell interactions and extravasation. For this reason, cancer cells use these endogenous mechanisms to stimulate an inflammatory milieu and release angiogenic factors that stimulate the endothelium to expose adhesion molecules, which enable adhesion and extravasation of proangiogenic monocytes (128). There are different markers described for these proangiogenic monocytes that cancer cells recruit to the TME. Different authors have found that human CD16^+^ patrolling monocytes promote angiogenesis and boost the expansion of human colorectal carcinoma xenografts (128, 129). These CD16^+^ monocytes seem to be recruited to the TME by cancer-released inflammatory cytokines and angiogenic factors (TNF-α, IL-1β, IL13, VEGF, etc.). In contrast, other investigations have described that inflammatory GR1^+^ monocytes are the proangiogenic monocyte subset that supports the growth of primary tumors (130, 131). In any case, once proangiogenic monocytes are attached to inflamed endothelium within the TME, these monocytes are locally retained by endothelial CX3CL1/Fractalkine released by ECs in response to interferon gamma (IFN-γ), which is present in the inflammatory locus (132). CX3CL1 is a distinctive CX3C chemokine that is anchored to the cell membrane to allow leucocyte adhesion (133, 134), although it is also present in a soluble form that exhibits monocyte and lymphocyte chemotaxis properties (135). Furthermore, CX3CL1 also facilitates vascular extravasation of monocytes in lung tumor metastasis (136).

Hypoxia is a driving force of angiogenesis in tumors (137, 138). Some investigations have described that hypoxia, *via* hypoxia-inducible factor-1 (HIF-1), enhances the expression of proangiogenic factors such as VEGF, Ang-1 and Ang-2 in endothelial and cancer cells (139, 140), and promotes the synthesis of CXCR4 (a receptor for CXCL12) and Tie2 (angiopoietin receptor) on macrophages, which allows the interstitial migration of proangiogenic macrophages inside the tumor (128, 141, 142). Numerous tumor-derived chemokines are critical for recruiting monocytes into the tumor milieu and to promote the transition of monocytes to tumor-associated macrophages (TAM). These include chemokines such as CCL3 (macrophage inflammatory protein, MIP1α), CCL2 (MCP-1) and CCL4 (MIP1β), interleukins (IL-6 and IL-1β) and cytokines (colony stimulating factor 1 (CSF-1) (143–145). Monocytes recruited into the tumor are then further reprogrammed by cancer cells to display a proangiogenic and immunotolerant M2-like macrophage phenotype (146). Indeed, tumor-associated macrophages (TAMs) are the most abundant immunosuppressive cells in the TME. They play a fundamental role in the tumor initiation, growth, and progression (147). M2-like TAM, under hypoxic milieu, also release proangiogenic factors such as VEGF and placental growth factor (PIGF), and chemokines such as CCL2 and CXCL9, that regulate the expansion of peritumoral vascular network (143, 145). Additionally, TAMs are also involved in immunosuppression of CD8^+^ T cells and natural killer (NK) cells, a major mechanism of anti-tumor immunity (148, 149).

The proangiogenic activity of TAM is also mediated by MMP activity, which contribute to the release of matrix-bound growth factors such as VEGF. For example, active MMP-9 is produced by mouse and human proangiogenic Tie2^+^ monocytes (150). The release of different matrix-bound growth factors from TME by active MMP-9 represents one of the most challenging mechanisms for current therapies to interfere with tumor angiogenesis (141). Indeed, digestion and delivery of angiogenic factors by MMPs is a vicious circle for tumor angiogenesis. Tumor tissues synthetize and accumulate MMPs and growth factors in the extracellular matrix, and simultaneously induce the arrival of more proangiogenic monocytes. Then, these monocytes infiltrate and transdifferentiate into TAM and secrete additional MMP-9 and growth factors to the TME, as suggested by previous studies in mice (151, 152).

Hypoxic TAMs show deep variations in the expression of numerous metabolic genes because they are compelled to adjust their metabolism to low oxygen pressure to maintain the energy needs (153). Cytokines are also important effectors on macrophage metabolism, inducing a wide array of metabolic changes. For example, pro-inflammatory M1-like macrophages change their metabolism toward increased anaerobic glycolysis, pentose phosphate pathway activation, and protein and fatty acid synthesis (154). In contrast, cytokines such as IL-4, IL-10, and IL-13 released by M2-like macrophages lead to a phenotype that more closely resembles the characteristics of TAMs (displaying enriched oxidative phosphorylation and stable glycolysis) (155). It is known that metabolic changes influence angiogenic and immunosuppressive properties of hypoxic TAMs. One signaling pathways governing these events is REDD1, a negative regulator of mTOR (156). Indeed, REDD1-mediated inhibition of mTOR hampers glycolysis in TAMs and reduces their excessive angiogenic response promoting the formation of anomalous blood vessels. Hence, TAMs lacking REDD1 promote the formation of smoothly aligned, pericyte-covered, functional vessels preventing vascular leakage, hypoxia, and metastases. TAMs deficient in REDD1 are highly glycolytic and drain glucose from TME affecting nearby ECs. This hinders vascular hyperactivation and stimulates the formation of quiescent vascular junctions (156). This functional link between TAM metabolism and tumor angiogenesis could be exploited in the future for the design of novel anti-angiogenic therapies for cancer.

## 4 Monocyte-endothelial cell interactions in tissue remodeling

### 4.1 Modulation of monocytes and MDM in atherosclerosis and cardiovascular diseases

#### 4.1.1 Monocytes and MDM in atherogenesis

**Figure 5:**
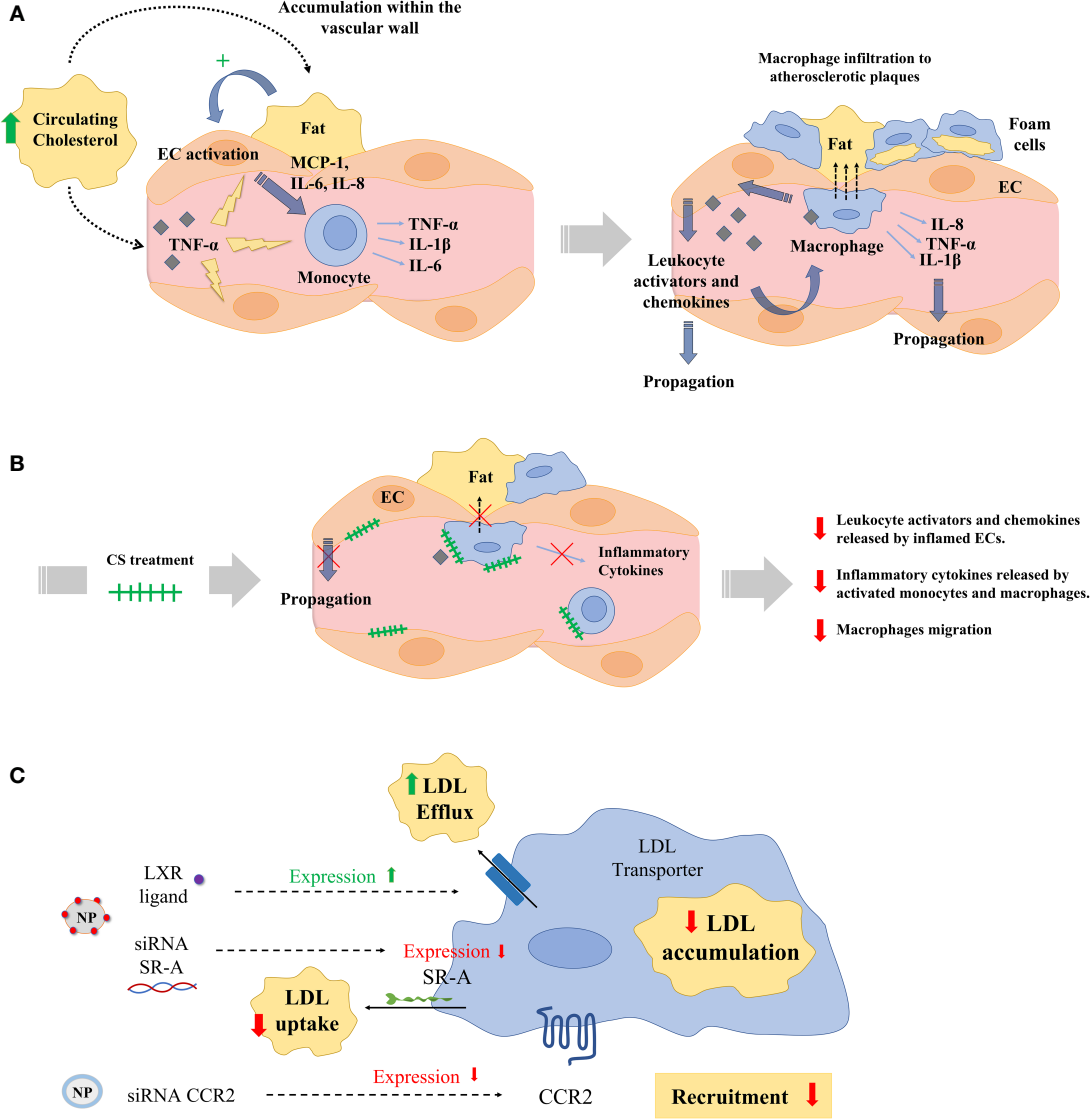
**(A)** Hypercholesterolemia, high blood pressure, or disrupted flow patterns lead to LDL accumulation within the vascular wall. This accumulation activates endothelial cells (ECs), which recruit and activate monocytes by the secretion of chemokines and monocyte activators (MCP-1, IL-6, IL-8). Then, monocytes infiltrate atherosclerotic lesions, differentiating into macrophages and ultimately into foam cells. Macrophages and foam cells deliver pro-inflammatory mediators (TNF-α, IL-1β), which decisively participate in the propagation of the inflammatory response and plaque progression. **(B)** Exogenous administration of CS disrupts the release of leukocyte activators and chemokines from aortic ECs inflamed with TNF-α and interferes with the release of inflammatory cytokines in activated monocytes and macrophages, and with their migration. **(C)** Numerous nanoparticles (NPs) have been used to target and treat macrophages in experimental atherosclerosis. Nanoparticles have aimed at reducing low density lipoprotein (LDL) accumulation in macrophages either reducing the macrophage expression of LDL scavenger receptors (SRs) using siRNA or delivering the liver-x-receptor (LXR) ligand to increase the expression of cholesterol transporters. Other NPs have been designed to deliver siRNA against the expression of the chemokine receptor 2 (CCR2) and reduce monocyte recruitment to atherosclerotic plaques.

CVDs are the leading cause of death and disability worldwide (157). The main etiological factor for CVD is atherosclerosis (158). Atherosclerosis predominantly affects the coronary circulation and increases the risk of myocardial infarction and stroke, but shear stress caused by atheroma plaques may also affect the peripheral and cerebrovascular circulation (159, 160). Atherosclerosis is currently understood as a chronic inflammatory disorder involving many immune cell types (161). Namely, MDM were the first inflammatory cells identified in the atherosclerotic plaques (162). Monocytes also contribute substantially to the different stages of atherosclerosis and myocardial infarction including initiation, progression, thrombus formation, and scarring (163, 164). Innate immune response is initially activated during early arterial injury, which induces the recruitment of bone marrow–derived monocytes into the intima (165). Hypercholesterolemia and clonal hematopoiesis of indeterminate potential are known risk factors in atherosclerosis *via* boosting the recruitment of mononuclear phagocytes into atheroma plaques (166). Monocytes that infiltrate into the intima of injured vessels transdifferentiate to inflammatory macrophages and can proliferate and promote plaque progression (**Figure 5A**). Monocyte and macrophage subsets secrete chemokines and cytokines that may stabilize or unstabilize the plaque (167). Generally, M1-like inflammatory macrophages increase the risk for unstable plaques and a class of M2-like resolving macrophages seem to promote plaque stabilization *via* efferocytosis, collagen production, and TGF-β production (168, 169). However, it is important to emphasize that there are multiple subpopulations of macrophages coexisting within the atheroma plaque with distinct genetic markers and functions, such as resident-like anti-inflammatory, inflammatory, and TREM2^high^ macrophages (170). ECs in injured vascular walls also release an array of chemokines that are commonly classified in four subgroups: CXC, CC, C, and CX3C chemokines. CX3CL1, also known as fractalkine, is the only member of the CX3C chemokine family. CX3CL1 and other chemokines such as CXCL12 may bind either to heparan sulfate (HS) on ECs or to their chemokine receptors in monocytes (171). Therefore, these chemokines may attract, retain, or induce monocyte transdifferentiation into macrophages and foam cells thereby displaying a major role on the progression of atheroma plaques and atherosclerosis (172, 173).

#### 4.1.2 Monocytes and MDM in angiogenesis and inflammation

Angiogenesis and inflammation are close collaborators in tissue remodeling following vascular injury and atherosclerosis. One of the initial signatures of early atherosclerosis is the appearance of a dysfunctional endothelium (174). Some of the factors affecting endothelial homeostasis include high levels of modified low-density lipoproteins (LDL), shear stress, free radicals, and hypertension (175). Indeed, dysfunctional endothelium induces an inflammatory response that may progress to a vascular lesion in CVDs or pave the ground for cancer metastasis (176). As described in the section 3 of this review, infiltrating inflammatory cells secrete a wide array of proangiogenic cytokines that stimulate EC activation, proliferation, and migration. However, the role of angiogenesis in atherosclerosis and CVDs is still a controversial and unresolved issue. Although proangiogenic therapy is useful for the treatment of ischemic heart disease and to enhance endothelial protective functions, angiogenesis may contribute to the growth of atherosclerotic lesions, and is a key factor in plaque destabilization and rupture at every vascular scale (177). The vasa vasorum, a specialized microvasculature that originates primarily in the adventitia of large arteries, is activated during atherosclerosis (178) and may well be influenced by MDM and EC-monocyte interactions. Vasa vasorum provides oxygen and nutrients to the external layers of the arterial wall and its expansion arises preceding endothelial dysfunction, intimal thickening, or plaque formation (178). The presence of intraplaque vasa vasorum is a marker of plaque expansion, progression, hemorrhage, instability, and rupture (178). In this scenario, proangiogenic molecules released by monocytes and MDM promote the growth of vasa vasorum and intimal lesions in both early and late stages of the disease. Moreover, the interactions between monocytes or MDM and ECs within atherosclerotic lesions may also be influenced by the local EC subpopulation. Indeed, analysis of the different EC subpopulations in aorta has identified a lymphatic EC cluster and two other populations specialized in lipoprotein handling, angiogenesis, and ECM production (179). Therefore, different subpopulations of monocytes and MDM may interact with distinct EC populations with vascular disease-relevant functions to decide the fate of the vascular lesion.

#### 4.1.3 Monocytes and MDM in other vascular alterations

There are other scenarios of vascular remodeling where monocytes and MDM display essential regulatory roles such as in pulmonary hypertension, thrombosis disorders, and venous malformation. Data obtained with single-cell analysis has revealed that non-classical and intermediate monocytes are enriched among all the monocyte subsets associated with pulmonary hypertension, and these phenotypes were associated with a decrease in hypoxia-inducible transcription factor-1α (HIF-1α) (180). Indeed, nonclassical (CD14^+^CD16^+^) monocytes sense hypoxia, modulate pulmonary vascular remodeling, and induce pulmonary hypertension (181). Moreover, the presence of perivascular MDM has emerged as a key pathogenic driver of pulmonary hypertension *via* interstitial macrophage-dependent inflammation and vascular remodeling (182). In thrombosis, monocytes and MDM release tissue factor, which activates prothrombin and initiates a coagulation cascade resulting in fibrin formation and thrombus formation (183). Monocytes and MDM also participate in the pathogenesis of venous malformation and cavernous venous malformation stimulating angiogenesis *via* VEGF overexpression in monocytes and MDMs and up-regulation of VEGF receptors in ECs (184).

#### 4.1.4 Monocytes and MDM in the regulation of vascular smooth muscle cells

Monocytes and MDM also modulate vascular smooth muscle cells (VSMCs) functions during atherosclerosis (185). The regulation of VSMC phenotypes may influence plaque morphology (necrotic core size and fibrous cap thickness) and the deposition and distribution of milieu components (lipoprotein, ECM, and chemokines). In turn, VSMCs, also interact with ECs to orchestrate response to injury or control EC growth (186, 187). VSMCs are major contributors to modifications of vascular microenvironment in CVDs by producing ECM proteins (e.g., fibrin, fibronectin, collagen, and proteoglycans) and agents that regulate ECM formation (e.g., tissue inhibitors of metalloproteinases, tissue factor). Alterations in some of these subendothelial matrix components influence EC apoptosis, which plays pivotal roles in atherosclerosis (188). Moreover, these variations in the ECM composition alter mechanical and functional properties of the vascular wall, which influence the behavior of monocytes, MDM, and ECs (189, 190). Namely, the interaction of the substratum component chondroitin sulfate (CS) has demonstrated a wide array of regulatory functions on monocytes/MDM and ECs in the context of inflammation and atherogenesis (191). In advanced atherosclerosis, there is a decrease in CS, with a concomitant increase of dermatan sulfate in arterial walls (192). Exogenous administration of CS disrupts the release of leukocyte activators and chemokines from aortic ECs inflamed with TNF-α and interferes with the release of inflammatory cytokines in activated monocytes and macrophages, and with their migration (**Figure 5B**) (191). Indeed, oral administration of CS to ApoE knockout mice has demonstrated to reduce the area of atheroma plaques *via* interference with the release of monocyte attractants and the uptake and accumulation of Ox-LDL in macrophages and foam cells (193). These findings have motivated a growing interest in incorporating either CS or other immunomodulator drugs in the composition of new intravascular devices for the treatment of CVDs (194–196).

#### 4.1.5 Therapeutic strategies to modulate monocytes and MDM in atherosclerosis and CVDs

Monocytes and MDM have a significant impact on lesion progression at all stages of atherogenesis, and there has been great effort in design novel therapies to interfere with the monocyte-EC or macrophage-atheroma plaque interactions in atherosclerosis (**Table 3**) (2, 161, 210). Standard therapies for atherosclerotic vascular disease (e.g. angiotensin converting enzyme inhibitors, aspirin, corticosteroids, etc.) induce general immune responses but not a specific macrophage targeting. Immunosuppressive therapies may display side effects in patients such as vulnerability to infection and cancer. A precise macrophage targeting seems essential for the treatment of atherosclerosis. Molecular signaling of peroxisome proliferator-activated receptors (PPARs) is of major relevance for the regulation of macrophage lipid metabolism and inflammatory responses. Natural ligands such as prostaglandins and anti-diabetic thiazolidinediones induce PPARs, which in turn stimulate the M2-like macrophage phenotype and a reduced progression of atherosclerosis (197). Liver X receptors are up-regulated in M2-like macrophages and, as occurs with PPARs, exert relevant antiatherosclerotic effects *via* regulation of cholesterol metabolism and M1-like macrophage inflammatory response (211). Statins are efficient cholesterol-reducing agents that also reduce immune responses *via* inhibition of macrophage inflammatory activity (212). However, these and other conventional pharmacological agents cannot selectively target macrophages. Numerous potential therapies based on nanoparticles (NPs) to target and treat macrophages have been used during the last decade to treat experimental atherosclerosis (**Figure 5C**). For example, a wide array of NPs functionalized with different targeting ligands such as mannose, hyaluronan, folate, DNA, peptides, antibodies, HDLs and LDLs have been employed to target intraplaque macrophages and to enhance selectivity and delivery of anti-inflammatory drugs to reduce atherosclerosis (213). Other strategies aim to reduce LDL accumulation in macrophages either reducing the macrophage expression of LDL scavenger receptors (SRs) or delivering the liver-x-receptor ligand to increase the expression of cholesterol transporters (198, 199). Other researchers have developed NPs to inhibit the expression of the chemokine receptor 2 (CCR2) associated with monocyte recruitment as a potential treatment for atherosclerosis (200, 201). Another interesting strategy for the treatment of atherosclerosis is the enhancement of macrophage efferocytosis – the phagocytic clearance of dying cells and apoptotic and necrotic debris. Indeed, this process is diminished in atherosclerotic blood vessels. Interestingly, NPs delivering siRNA against Ca^2+^/calmodulin-dependent protein kinase γ (a known blocker of macrophage efferocytosis) have shown plaque stabilization in mice (202). Therefore, it seems clear that specific modulation of macrophage interactions and functions in vascular lesions using targeted nanoparticles may be of therapeutic interest for the treatment of atherosclerosis and CVDs in the future.

**Table 3:** Novel therapies to modulate monocytes and MDM.

Treatment	Target cell	Disease	Treatment strategy	Reference
**Chondroitin sulfate (CS)**	ECs Monocytes and macrophages	Atherosclerosis	Disruption of the propagation of inflammation	(193)
**Prostaglandins** **Anti-diabetic thiazolidinediones**	Macrophages	Atherosclerosis	Stimulation of the M2-like macrophage phenotype	(197)
**NPs with siRNA against LDL scavenger receptors**	Macrophages	Atherosclerosis	Reduction of LDL accumulation	(198, 199)
**NPs with Liver-x-receptor ligand**	Macrophages	Atherosclerosis	Reduction of LDL accumulation	(198, 199)
**NPs with siRNA against CCR2**	Macrophages	Atherosclerosis	Reduction of monocyte recruitment	(200, 201)
**NPs with siRNA against Ca2+/calmodulin-dependent protein kinase γ**	Macrophages	Atherosclerosis	Enhancement of macrophage efferocytosis	(202)
**PLGA-NPs with SYK pathway inhibitor**	Macrophages	Steatohepatitis	Inhibition of inflammatory pathways	(203)
**Liposomes loaded with dexamethasone**	Macrophages	Acute and chronic liver diseases	Induction of anti-inflammation	(204)
**Dendrimer-graphene nanostars with PPAR-γ agonist GW1929**	Macrophages	Hepatic fibrosis	Reduce Hepatic inflammation and fibrosis	(205)
**Nanostructured lipid carriers containing curcumin**	Macrophages	Hepatic fibrosis	Reduction of hepatic inflammation	(206)
**Dendrimer-graphene nanostars containing cDNA MMP9**	Macrophages	Hepatic fibrosis	Local digestion of collagen fibers	(207)
**CXCR4-targeted lipid-coated PLGA NPs with sorafenib and AMD3100**	TAM	Hepatocellular carcinoma	Reduction of M2-like macrophage polarization and TAMs infiltration	(208)
**NPs with mRNA encoding BisCCL2/5i**	TAM	Hepatocellular carcinoma	Induction of macrophages polarization to antitumoral M1-like subtype	(209)

### 4.2 Modulation of monocytes and MDM in chronic liver diseases

#### 4.2.1 Contribution of monocytes and MDM to the pathogenesis of chronic liver diseases

Chronic liver injury from different etiologies may lead to hepatic fibrosis, cirrhosis, and/or hepatocellular carcinoma (HCC). Cirrhosis is among the most prevalent diseases in Western countries. The prognosis of these patients is grim, except for those who can benefit from liver transplantation. This is due to the multiple organic derangements, including renal failure, variceal bleeding, or bacterial peritonitis (214). These patients develop an important and progressively accentuated cardiocirculatory dysfunction, portal hypertension, arterial hypotension, high cardiac output, and diminished systemic vascular resistance. It is commonly assumed that increased endothelial production of NO is of major importance in the pathogenesis of the circulatory dysfunction occurring in cirrhosis (215), but the contribution of other endogenous systems has also been considered (216). In this scenario, MDM also contribute to vascular dysfunction in cirrhosis *via* release of vasodilators such as NO or adrenomedullin (217, 218). Hypoxia appears as a common phenomenon associated with the induction and release of NO from macrophages during liver cirrhosis (219). Another general activator of macrophages in liver cirrhosis is bacterial lipopolysaccharide (LPS). Intestinal permeability is increased in patients with advanced liver cirrhosis, and bacterial translocation may cause infection and spontaneous bacterial peritonitis that usually results in renal failure and death despite the efficacy of the antibiotic therapy (214). Macrophages isolated from cirrhotic patients with bacterial peritonitis have shown a high release of VEGF that results in increased vascular permeability in the peritoneal vessels of these patients (220). LPSs also suppress the expression of the cannabinoid receptor CB2 in circulating monocytes and peritoneal macrophages, impairing the defense response mechanisms in cirrhotic patients with liver disease (221). Macrophage CB2 activation has also been associated with reduced angiogenesis attributed to a lower monocyte infiltration during liver fibrosis and a lesser macrophage release of proangiogenic factors (222, 223).

#### 4.2.2 Role of monocytes and MDM on liver regeneration

MDM are critical players during all the stages of chronic liver injury: initiation, progression, resolution, and regeneration of the hepatic function (224). Indeed, the liver is a unique organ in its capacity to regenerate the entire organ mass after a liver insult or a hepatic resection (225). After liver resection, injured hepatocytes, liver progenitor cells and KCs from the portal space recruit circulating monocytes to injured liver *via* secretion of monocyte chemotactic protein 1 (MCP-1) (226). Then, recruited monocytes trigger endothelial c-Met and Tie2 pathways by direct interaction (92–94), and activate the paracrine release of different cytokines and endothelial growth factors critical for liver regeneration, such as the family of molecules Notch and Wnt (91, 227). Indeed, either complete depletion of hepatic macrophages or interference of the canonical Wnt/β-catenin signaling pathway in macrophages reduces liver regeneration (228, 229). KCs elaborate a precise control role of sinusoidal endothelium releasing priming factors (e.g., IL-6 and TNF-α) and induce hepatocytes to act in response to growth factors (e.g., HGF, TGF-β, and EGF). Then, proliferation of hepatocytes sequentially advances from periportal to pericentral areas of the lobule, as a wave of mitosis under circadian control. In turn, hepatocyte proliferation needs to be perfectly coordinated with the expansion of the hepatic vascular network during liver regeneration (230). A previous work has described how this sequence of phenomena is fine-tune regulated by monocytes and MDM in regeneration occurring after liver resection. It has been demonstrated that circulating monocytes are selectively recruited to sprouting spots in regenerating livers. This process starts in portal areas and expands to the rest of hepatic tissue coinciding with the waves of hepatocyte mitosis, the hepatic expression of iNOS and vasodilation to facilitate monocyte infiltration and endothelial migration as explained in section 3.1 of this review. These interactions between monocytes and hepatic ECs commensurate with phosphorylation and disruption of VE-cadherin connections, which is crucial for endothelial tip migration and elongation (231).

MDM display different roles in liver regeneration initiated after chronic injury and fibrosis. MDMs that infiltrate during the progression of liver fibrosis propagate inflammation and induce scarring *via* activation of myofibroblasts (232). The balance between stimuli from microenvironment and the presence of different subsets of macrophages is determinant towards liver disease or repair (233). Recent advances in flow cytometry and single-cell transcriptomics have allowed a broad understanding of the array of macrophage phenotypes within healthy and diseased liver (234). Nowadays, these technologies have allowed the determination of different subsets of mononuclear phagocytes in the liver fibrotic niche, which is composed of ten main clusters: scar-associated macrophages (SAMacs), KCs, tissue monocytes (TMs), conventional dendritic cells (cDCs) and each corresponding cycling (proliferating) cell subsets (234). SAMacs express the unique markers TREM2 and CD9 (234). These macrophages displayed a hybrid phenotype, between TMs and KCs and similar to MDM in mouse liver injury models. SAMacs have a pro-fibrogenic phenotype and accumulate within the fibrotic niche in cirrhotic liver. TREM2^+^CD9^+^ SAMacs have showed a monocyte-like morphology and a distinctive topographical distribution and differentiation trajectory separated from KCs (234).

#### 4.2.3 Monocytes and MDM on liver cancer

Liver cancer is the third leading cause of cancer-related mortality worldwide and HCC accounts for nearly 90% of the incidence of all hepatic cancers (235). The tumor microenvironment (TME) is composed by a highly complex cellular composition including different populations of myeloid cells and lymphocytes. Indeed, the presence of myeloid cells in the TME is frequently associated to altered patient survival (236). In this scenario, TAM is one of the main members of the TME with a critical role on HCC occurrence and development *via* angiogenesis stimulation, interference with T cell anticancer activity, promotion of drug resistance, and cancer metastasis (148, 237). The state-of-the-art combination of two single-cell RNA sequencing technologies has recently allowed the analysis of all CD45^+^ immune cells in HCC patients from different hepatic zones (tumor, adjacent liver, hepatic lymph node, blood, and tumor ascites) (238). In this study, TAMs were associated with poor prognosis and these macrophages highly expressed two marker genes, SLC40A1 and GPNMB, in HCC tumors. Namely, SLC40A1 encodes ferroportin, an iron exporter, and regulates TLR-stimulus-induced pro-inflammatory cytokines, including IL-6, IL-23, and IL-1β, thus pointing out that iron metabolism is implicated in determining innate immunity in the TME.

#### 4.2.4 Therapeutic strategies to modulate monocytes and MDM in chronic liver diseases

Cirrhosis is a limiting factor for anticancer therapy and a major risk factor for the development of HCC. Indeed, cirrhosis may challenge surgical and interventional approaches to treat liver cancer, alter pharmacokinetics of anticancer drugs, enhance side effects of chemotherapeutics and susceptibility to hepatotoxicity. Therefore, conventional and future treatments designed to modulate monocytes and MDM in liver diseases need to consider the different molecular signals involved during liver cirrhosis (M1-like inflammatory macrophages and fibrogenic MDM signals) or liver cancer (anti-inflammatory M2-like TAM in the TME and immunosuppression). Various strategies have been developed to treat liver inflammation and to target hepatic macrophages (**Table 3**) (239). For example, polylactic-co-glycolic acid (PLGA) NPs with a Spleen Tyrosine kinase (SYK) pathway inhibitor have been used to target and treat macrophages in chronic liver injury induced by steatohepatitis, since SYK is a critical mediator in inflammatory pathways (203). Liposomes loaded with the anti-inflammatory drug dexamethasone have obtained anti-inflammatory and anti-fibrotic results in mouse models of acute and chronic liver disease (204). A PPAR-γ agonist GW1929 targeted to MDM with dendrimer-graphene nanostars has been used to reduce hepatic inflammation and fibrosis (205). Phosphatidylserine (a component that mimics apoptotic cells recognized by macrophages) has been used to decorate nanostructured lipid carriers containing curcumin to reduce hepatic inflammation and fibrosis (206). Another interesting approach has been the delivery of a plasmid expressing the collagenase metalloproteinase 9 into inflammatory macrophages using dendrimer-graphene nanostars for local digestion of collagen fibers, reduction of hepatic injury, and hepatic regeneration (207). Different strategies have also been developed to target and treat selectively TAM in HCC (240). For example, CXCR4-targeted lipid-coated PLGA NPs with sorafenib and AMD3100 (a CXCR4 antagonist) revealed that blocking the interaction of TAM CXCR4 with SDF1α reduced M2-like macrophage polarization and TAMs infiltration, and simultaneously tumor progression was delayed in a mouse model of HCC (208). CCL2 and CCL5 are two chemokines that attract TAMs infiltration and induce their polarization to the M2-like phenotype. A specific CCL2/CCL5 dual inhibitor (BisCCL2/5i) coating lipid NPs or a mRNA encoding BisCCL2/5i inhibited TAM infiltration and induced the polarization of M2-like macrophages to antitumoral M1-like subtype (209). Overall, further studies are still necessary to delineate how to combine conventional therapies against HCC cells with TAM-targeted nanotherapeutics to overcome the limitations of current pharmacological treatments in HCC.

## 5 Conclusions

This review has emphasized that interactions between monocytes and ECs or between transmigrated and differentiated MDM and the milieu are critical to the orchestration of vascular and tissue remodeling. The interplay between monocytes and ECs determines the selection of sprouting points during angiogenesis and may be involved in vasculogenesis. Infiltrated MDM regulate the promotion of vascular and tissue growth *via* the release of different vascular growth factors, regulation of vascular permeability, and control over ECM turnover. Tumors take advantage of the physiological functions of proangiogenic monocytes and MDM to increase cancer cell irrigation and metastasis.

Blood monocytes and MDM play a critical role in the pathogenesis of myocardial infarctions, strokes, and CVDs overall. They actively contribute to new vessel formation inside the arterial wall and atherosclerotic plaques resulting in local ischemia and inflammation, and regulate the activity and phenotype of VSMC, thereby influencing plaque morphology and ECM deposition.

Chronic liver diseases are characterized by a vigorous activation of inflammatory subsets of monocytes and MDM that mediate hepatic angiogenesis, inflammation, and fibrosis. Monocytes and MDM display different roles during regeneration of healthy liver or after chronic injury and fibrosis. Monocyte-EC interactions orchestrate harmonized angiogenesis and synchronized liver mass growth after resection in healthy liver. In contrast, monocytes and MDM perpetuate angiogenesis and inflammation in chronic liver diseases and pave the ground for hepatic tumor growth.

Conventional and novel therapeutic strategies are being developed to selectively target monocytes and MDM to modulate the progression of chronic diseases such as CVD or liver diseases, and cancer. There is still an urgent need for more selective treatments and molecular insights on the different macrophage stirpes involved in the zonation phenomena occurring during vascular and tissue remodeling, and cancer. It is mandatory to decipher surface markers and mechanisms involved in the control of specific subsets of monocytes and MDM to improve therapeutic interventions without distortion of the necessary physiological functions of these immune cells in pathogen detection and tissue regeneration.

## Author contributions

MM-B, BS-C, PM-L: Drafting of the manuscript and preparation of tables and figures. ERE and WJ: Revision of the manuscript. All authors contributed to the article and approved the submitted version.

## References

[1] Ginhoux F Jung S. Monocytes and macrophages: developmental pathways and tissue homeostasis. Nat Rev Immunol (2014) 14:392–404. doi: 10.1038/nri3671 24854589

[2] Medrano-Bosch M Moreno-Lanceta A Melgar-Lesmes P. Nanoparticles to target and treat macrophages: the ockham’s concept? Pharmaceutics (2021) 13(9):1340. doi: 10.3390/pharmaceutics13091340 34575416 PMC8469871

[3] He W Kapate N Shields C Mitragotri S. Drug delivery to macrophages: a review of targeting drugs and drug carriers to macrophages for inflammatory diseases. Adv Drug Delivery Rev (2020) 165-166:15–40. doi: 10.1016/j.addr.2019.12.001 31816357

[4] Wang L Lu Q Gao W Yu S. Recent advancement on development of drug-induced macrophage polarization in control of human diseases. Life Sci (2021) 284:119914. doi: 10.1016/j.lfs.2021.119914 34453949

[5] Hashimoto D Chow A Noizat C Teo P Beasley MB Leboeuf M. Tissue-resident macrophages self-maintain locally throughout adult life with minimal contribution from circulating monocytes. Immunity (2013) 38:792–804. doi: 10.1016/j.immuni.2013.04.004 23601688 PMC3853406

[6] Yona S Kim KW Wolf Y Mildner A Varol D Breker M. Fate mapping reveals origins and dynamics of monocytes and tissue macrophages under homeostasis. Immunity (2013) 38:79–91. doi: 10.1016/j.immuni.2012.12.001 23273845 PMC3908543

[7] van Furth R Sluiter W. Distribution of blood monocytes between a marginating and a circulating pool. J Exp Med (1986) 163:474–9. doi: 10.1084/jem.163.2.474 PMC2188035 3944542

[8] Swirski FK Nahrendorf M Etzrodt M Wildgruber M Cortez-Retamozo V Panizzi P. Identification of splenic reservoir monocytes and their deployment to inflammatory sites. Science (2009) 325:612–6. doi: 10.1126/science.1175202 PMC2803111 19644120

[9] Parihar A Eubank TD Doseff AI. Monocytes and macrophages regulate immunity through dynamic networks of survival and cell death. J Innate Immun (2010) 2:204–15. doi: 10.1159/000296507 PMC2956013 20375558

[10] Mestas J Ley K. Monocyte-endothelial cell interactions in the development of atherosclerosis. Trends Cardiovasc Med (2008) 18:228–32. doi: 10.1016/j.tcm.2008.11.004 PMC2650852 19185814

[11] Bilzer M Roggel F Gerbes AL. Role of kupffer cells in host defense and liver disease. Liver Int (2006) 26:1175–86. doi: 10.1111/j.1478-3231.2006.01342.x 17105582

[12] Theurl I Hilgendorf I Nairz M Tymoszuk P Haschka D Asshoff M. On-demand erythrocyte disposal and iron recycling requires transient macrophages in the liver. Nat Med (2016) 22:945–51. doi: 10.1038/nm.4146 PMC4957133 27428900

[13] Krenkel O Tacke F. Liver macrophages in tissue homeostasis and disease. Nat Rev Immunol (2017) 17:306–21. doi: 10.1038/nri.2017.11 28317925

[14] Kono H Rock KL. How dying cells alert the immune system to danger. Nat Rev Immunol (2008) 8:279–89. doi: 10.1038/nri2215 PMC2763408 18340345

[15] Murray PJ Wynn TA. Protective and pathogenic functions of macrophage subsets. Nat Rev Immunol (2011) 11:723–37. doi: 10.1038/nri3073 PMC3422549 21997792

[16] Wynn TA Chawla A Pollard JW. Macrophage biology in development, homeostasis and disease. Nature (2013) 496:445–55. doi: 10.1038/nature12034 PMC3725458 23619691

[17] Wynn TA Vannella KM. Macrophages in tissue repair, regeneration, and fibrosis. Immunity (2016) 44:450–62. doi: 10.1016/j.immuni.2016.02.015 PMC4794754 26982353

[18] Melgar-Lesmes P Tugues S Ros J Fernandez-Varo G Morales-Ruiz M Rodes J. Vascular endothelial growth factor and angiopoietin-2 play a major role in the pathogenesis of vascular leakage in cirrhotic rats. Gut (2009) 58:285–92. doi: 10.1136/gut.2008.155028 18978178

[19] Yang J Antin P Berx G Blanpain C Brabletz T Bronner M. Guidelines and definitions for research on epithelial-mesenchymal transition. Nat Rev Mol Cell Biol (2020) 21:341–52. doi: 10.1038/s41580-020-0237-9 PMC7250738 32300252

[20] Ribera J Pauta M Melgar-Lesmes P Cordoba B Bosch A Calvo M. A small population of liver endothelial cells undergoes endothelial-to-mesenchymal transition in response to chronic liver injury. Am J Physiol Gastrointest Liver Physiol (2017) 313:G492–504. doi: 10.1152/ajpgi.00428.2016 28798084

[21] Waidmann O Brunner F Herrmann E Zeuzem S Piiper A Kronenberger B. Macrophage activation is a prognostic parameter for variceal bleeding and overall survival in patients with liver cirrhosis. J Hepatol (2013) 58:956–61. doi: 10.1016/j.jhep.2013.01.005 23333526

[22] Barrett TJ. Macrophages in atherosclerosis regression. Arterioscler Thromb Vasc Biol (2020) 40:20–33. doi: 10.1161/ATVBAHA.119.312802 31722535 PMC6946104

[23] Shi C Pamer EG. Monocyte recruitment during infection and inflammation. Nat Rev Immunol (2011) 11:762–74. doi: 10.1038/nri3070 PMC3947780 21984070

[24] Imhof BA Aurrand-Lions M. Adhesion mechanisms regulating the migration of monocytes. Nat Rev Immunol (2004) 4:432–44. doi: 10.1038/nri1375 15173832

[25] Geissmann F Jung S Littman DR. Blood monocytes consist of two principal subsets with distinct migratory properties. Immunity (2003) 19:71–82. doi: 10.1016/S1074-7613(03)00174-2 12871640

[26] Kapellos TS Bonaguro L Gemund I Reusch N Saglam A Hinkley ER. Human monocyte subsets and phenotypes in major chronic inflammatory diseases. Front Immunol (2019) 10:2035. doi: 10.3389/fimmu.2019.02035 31543877 PMC6728754

[27] Boyette LB Macedo C Hadi K Elinoff BD Walters JT Ramaswami B. Phenotype, function, and differentiation potential of human monocyte subsets. PloS One (2017) 12:e0176460. doi: 10.1371/journal.pone.0176460 28445506 PMC5406034

[28] Kratofil RM Kubes P Deniset JF. Monocyte conversion during inflammation and injury. Arterioscler Thromb Vasc Biol (2017) 37:35–42. doi: 10.1161/ATVBAHA.116.308198 27765768

[29] Charo IF Ransohoff RM. The many roles of chemokines and chemokine receptors in inflammation. N Engl J Med (2006) 354:610–21. doi: 10.1056/NEJMra052723 16467548

[30] Petermann M Orfanos Z Sellau J Gharaibeh M Lotter H Fleischer B. CCR2 deficiency impairs Ly6C(lo) and Ly6C(hi) monocyte responses in orientia tsutsugamushi infection. Front Immunol (2021) 12:670219. doi: 10.3389/fimmu.2021.670219 34290699 PMC8287586

[31] Tsou CL Peters W Si Y Slaymaker S Aslanian AM Weisberg SP. Critical roles for CCR2 and MCP-3 in monocyte mobilization from bone marrow and recruitment to inflammatory sites. J Clin Invest (2007) 117:902–9. doi: 10.1172/JCI29919 PMC1810572 17364026

[32] Jia T Serbina NV Brandl K Zhong MX Leiner IM Charo IF. Additive roles for MCP-1 and MCP-3 in CCR2-mediated recruitment of inflammatory monocytes during listeria monocytogenes infection. J Immunol (2008) 180:6846–53. doi: 10.4049/jimmunol.180.10.6846 PMC2386263 18453605

[33] Ranjbar M Rahimi A Baghernejadan Z Ghorbani A Khorramdelazad H. Role of CCL2/CCR2 axis in the pathogenesis of COVID-19 and possible treatments: all options on the table. Int Immunopharmacol (2022) 113:109325. doi: 10.1016/j.intimp.2022.109325 36252475 PMC9561120

[34] Landsman L Bar-On L Zernecke A Kim KW Krauthgamer R Shagdarsuren E. CX3CR1 is required for monocyte homeostasis and atherogenesis by promoting cell survival. Blood (2009) 113:963–72. doi: 10.1182/blood-2008-07-170787 18971423

[35] Mack M Cihak J Simonis C Luckow B Proudfoot AE Plachy J. Expression and characterization of the chemokine receptors CCR2 and CCR5 in mice. J Immunol (2001) 166:4697–704. doi: 10.4049/jimmunol.166.7.4697 11254730

[36] Weber C Weber KS Klier C Gu S Wank R Horuk R. Specialized roles of the chemokine receptors CCR1 and CCR5 in the recruitment of monocytes and T(H)1-like/CD45RO(+) T cells. Blood (2001) 97:1144–6. doi: 10.1182/blood.V97.4.1144 11159551

[37] Braunersreuther V Zernecke A Arnaud C Liehn EA Steffens S Shagdarsuren E. Ccr5 but not Ccr1 deficiency reduces development of diet-induced atherosclerosis in mice. Arterioscler Thromb Vasc Biol (2007) 27:373–9. doi: 10.1161/01.ATV.0000253886.44609.ae 17138939

[38] Zernecke A Liehn EA Gao JL Kuziel WA Murphy PM Weber C. Deficiency in CCR5 but not CCR1 protects against neointima formation in atherosclerosis-prone mice: involvement of IL-10. Blood (2006) 107:4240–3. doi: 10.1182/blood-2005-09-3922 16467202

[39] Anderson MW Zhao S Ai WZ Tibshirani R Levy R Lossos IS. C-c chemokine receptor 1 expression in human hematolymphoid neoplasia. Am J Clin Pathol (2010) 133:473–83. doi: 10.1309/AJCP1TA3FLOQTMHF PMC4305436 20154287

[40] Rot A von Andrian UH. Chemokines in innate and adaptive host defense: basic chemokinese grammar for immune cells. Annu Rev Immunol (2004) 22:891–928. doi: 10.1146/annurev.immunol.22.012703.104543 15032599

[41] Cyster JG. Chemokines, sphingosine-1-phosphate, and cell migration in secondary lymphoid organs. Annu Rev Immunol (2005) 23:127–59. doi: 10.1146/annurev.immunol.23.021704.115628 15771568

[42] Manthey HD Cochain C Barnsteiner S Karshovska E Pelisek J Koch M. CCR6 selectively promotes monocyte mediated inflammation and atherogenesis in mice. Thromb Haemost (2013) 110:1267–77. doi: 10.1160/TH13-01-0017 24114205

[43] Kling JC Mack M Korner H. The absence of CCR7 results in dysregulated monocyte migration and immunosuppression facilitating chronic cutaneous leishmaniasis. PloS One (2013) 8:e79098. doi: 10.1371/journal.pone.0079098 24205367 PMC3813618

[44] Gombert M Dieu-Nosjean MC Winterberg F Bunemann E Kubitza RC Da Cunha L. CCL1-CCR8 interactions: an axis mediating the recruitment of T cells and langerhans-type dendritic cells to sites of atopic skin inflammation. J Immunol (2005) 174:5082–91. doi: 10.4049/jimmunol.174.8.5082 15814739

[45] Wang H Shao Q Wang J Zhao L Wang L Cheng Z. Decreased CXCR2 expression on circulating monocytes of colorectal cancer impairs recruitment and induces re-education of tumor-associated macrophages. Cancer Lett (2022) 529:112–25. doi: 10.1016/j.canlet.2022.01.004 34999169

[46] Le Borgne M Etchart N Goubier A Lira SA Sirard JC van Rooijen N. Dendritic cells rapidly recruited into epithelial tissues *via* CCR6/CCL20 are responsible for CD8+ T cell crosspriming in vivo. Immunity (2006) 24:191–201. doi: 10.1016/j.immuni.2006.01.005 16473831

[47] Ravindran R Rusch L Itano A Jenkins MK McSorley SJ. CCR6-dependent recruitment of blood phagocytes is necessary for rapid CD4 T cell responses to local bacterial infection. Proc Natl Acad Sci U.S.A. (2007) 104:12075–80. doi: 10.1073/pnas.0701363104 PMC1907313 17615242

[48] Ivetic A Hoskins Green HL Hart SJ. L-selectin: a major regulator of leukocyte adhesion, migration and signaling. Front Immunol (2019) 10:1068. doi: 10.3389/fimmu.2019.01068 31139190 PMC6527602

[49] Xu H Manivannan A Crane I Dawson R Liversidge J. Critical but divergent roles for CD62L and CD44 in directing blood monocyte trafficking *in vivo* during inflammation. Blood (2008) 112:1166–74. doi: 10.1182/blood-2007-06-098327 PMC2515150 18391078

[50] Leon B Ardavin C. Monocyte migration to inflamed skin and lymph nodes is differentially controlled by l-selectin and PSGL-1. Blood (2008) 111:3126–30. doi: 10.1182/blood-2007-07-100610 18184867

[51] Nijhuis MM Pasterkamp G Sluis NI de Kleijn DP Laman JD Ulfman LH. Peptidoglycan increases firm adhesion of monocytes under flow conditions and primes monocyte chemotaxis. J Vasc Res (2007) 44:214–22. doi: 10.1159/000100420 17337907

[52] Sperandio M Smith ML Forlow SB Olson TS Xia L McEver RP. P-selectin glycoprotein ligand-1 mediates l-selectin-dependent leukocyte rolling in venules. J Exp Med (2003) 197:1355–63. doi: 10.1084/jem.20021854 PMC2193782 12756271

[53] Walcheck B Moore KL McEver RP Kishimoto TK. Neutrophil-neutrophil interactions under hydrodynamic shear stress involve l-selectin and PSGL-1. a mechanism that amplifies initial leukocyte accumulation of p-selectin *in vitro* . J Clin Invest (1996) 98:1081–7. doi: 10.1172/JCI118888 PMC507527 8787668

[54] Shimonaka M Katagiri K Nakayama T Fujita N Tsuruo T Yoshie O. Rap1 translates chemokine signals to integrin activation, cell polarization, and motility across vascular endothelium under flow. J Cell Biol (2003) 161:417–27. doi: 10.1083/jcb.200301133 PMC2172897 12707305

[55] Bos JL de Rooij J Reedquist KA. Rap1 signalling: adhering to new models. Nat Rev Mol Cell Biol (2001) 2:369–77. doi: 10.1038/35073073 11331911

[56] Lomakina EB Waugh RE. Signaling and dynamics of activation of LFA-1 and mac-1 by immobilized IL-8. Cell Mol Bioeng (2010) 3:106–16. doi: 10.1007/s12195-009-0099-x PMC3084010 21532911

[57] Ostermann G Weber KS Zernecke A Schroder A Weber C. JAM-1 is a ligand of the beta(2) integrin LFA-1 involved in transendothelial migration of leukocytes. Nat Immunol (2002) 3:151–8. doi: 10.1038/ni755 11812992

[58] Cunningham SA Rodriguez JM Arrate MP Tran TM Brock TA. JAM2 interacts with alpha4beta1. facilitation by JAM3. J Biol Chem (2002) 277:27589–92. doi: 10.1074/jbc.C200331200 12070135

[59] Santoso S Sachs UJ Kroll H Linder M Ruf A Preissner KT. The junctional adhesion molecule 3 (JAM-3) on human platelets is a counterreceptor for the leukocyte integrin mac-1. J Exp Med (2002) 196:679–91. doi: 10.1084/jem.20020267 PMC2194005 12208882

[60] Muller WA. Leukocyte-endothelial-cell interactions in leukocyte transmigration and the inflammatory response. Trends Immunol (2003) 24:327–34. doi: 10.1016/S1471-4906(03)00117-0 12810109

[61] Schenkel AR Mamdouh Z Chen X Liebman RM Muller WA. CD99 plays a major role in the migration of monocytes through endothelial junctions. Nat Immunol (2002) 3:143–50. doi: 10.1038/ni749 11812991

[62] McEver RP. Selectins: lectins that initiate cell adhesion under flow. Curr Opin Cell Biol (2002) 14:581–6. doi: 10.1016/S0955-0674(02)00367-8 12231353

[63] Fuhlbrigge RC Alon R Puri KD Lowe JB Springer TA. Sialylated, fucosylated ligands for l-selectin expressed on leukocytes mediate tethering and rolling adhesions in physiologic flow conditions. J Cell Biol (1996) 135:837–48. doi: 10.1083/jcb.135.3.837 PMC2121069 8909555

[64] Lowe JB. Glycan-dependent leukocyte adhesion and recruitment in inflammation. Curr Opin Cell Biol (2003) 15:531–8. doi: 10.1016/j.ceb.2003.08.002 14519387

[65] Gerhardt T Ley K. Monocyte trafficking across the vessel wall. Cardiovasc Res (2015) 107:321–30. doi: 10.1093/cvr/cvv147 PMC4592323 25990461

[66] Parish CR. The role of heparan sulphate in inflammation. Nat Rev Immunol (2006) 6:633–43. doi: 10.1038/nri1918 16917509

[67] Laudanna C Kim JY Constantin G Butcher E. Rapid leukocyte integrin activation by chemokines. Immunol Rev (2002) 186:37–46. doi: 10.1034/j.1600-065X.2002.18604.x 12234360

[68] Bos JL. Linking rap to cell adhesion. Curr Opin Cell Biol (2005) 17:123–8. doi: 10.1016/j.ceb.2005.02.009 15780587

[69] Katagiri K Maeda A Shimonaka M Kinashi T. RAPL, a Rap1-binding molecule that mediates Rap1-induced adhesion through spatial regulation of LFA-1. Nat Immunol (2003) 4:741–8. doi: 10.1038/ni950 12845325

[70] Lafuente EM van Puijenbroek AA Krause M Carman CV Freeman GJ Berezovskaya A. RIAM, an Ena/VASP and profilin ligand, interacts with Rap1-GTP and mediates Rap1-induced adhesion. Dev Cell (2004) 7:585–95. doi: 10.1016/j.devcel.2004.07.021 15469846

[71] Han J Lim CJ Watanabe N Soriani A Ratnikov B Calderwood DA. Reconstructing and deconstructing agonist-induced activation of integrin alphaIIbbeta3. Curr Biol (2006) 16:1796–806. doi: 10.1016/j.cub.2006.08.035 16979556

[72] Calderwood DA. Integrin activation. J Cell Sci (2004) 117:657–66. doi: 10.1242/jcs.01014 14754902

[73] Hynes RO. Integrins: bidirectional, allosteric signaling machines. Cell (2002) 110:673–87. doi: 10.1016/S0092-8674(02)00971-6 12297042

[74] Rose DM Alon R Ginsberg MH. Integrin modulation and signaling in leukocyte adhesion and migration. Immunol Rev (2007) 218:126–34. doi: 10.1111/j.1600-065X.2007.00536.x 17624949

[75] Sanchez-Madrid F del Pozo MA. Leukocyte polarization in cell migration and immune interactions. EMBO J (1999) 18:501–11. doi: 10.1093/emboj/18.3.501 PMC1171143 9927410

[76] Coates TD Watts RG Hartman R Howard TH. Relationship of f-actin distribution to development of polar shape in human polymorphonuclear neutrophils. J Cell Biol (1992) 117:765–74. doi: 10.1083/jcb.117.4.765 PMC2289466 1577856

[77] Kinashi T. Intracellular signalling controlling integrin activation in lymphocytes. Nat Rev Immunol (2005) 5:546–59. doi: 10.1038/nri1646 15965491

[78] Plant PJ Fawcett JP Lin DC Holdorf AD Binns K Kulkarni S. A polarity complex of mPar-6 and atypical PKC binds, phosphorylates and regulates mammalian lgl. Nat Cell Biol (2003) 5:301–8. doi: 10.1038/ncb948 12629547

[79] Luscinskas FW Ma S Nusrat A Parkos CA Shaw SK. Leukocyte transendothelial migration: a junctional affair. Semin Immunol (2002) 14:105–13. doi: 10.1006/smim.2001.0347 11978082

[80] Bazzoni G. The JAM family of junctional adhesion molecules. Curr Opin Cell Biol (2003) 15:525–30. doi: 10.1016/S0955-0674(03)00104-2 14519386

[81] Bradfield PF Scheiermann C Nourshargh S Ody C Luscinskas FW Rainger GE. JAM-c regulates unidirectional monocyte transendothelial migration in inflammation. Blood (2007) 110:2545–55. doi: 10.1182/blood-2007-03-078733 PMC1988941 17625065

[82] Sullivan DP Muller WA. Neutrophil and monocyte recruitment by PECAM, CD99, and other molecules *via* the LBRC. Semin Immunopathol (2014) 36:193–209. doi: 10.1007/s00281-013-0412-6 24337626 PMC3991761

[83] Hixenbaugh EA Goeckeler ZM Papaiya NN Wysolmerski RB Silverstein SC Huang AJ. Stimulated neutrophils induce myosin light chain phosphorylation and isometric tension in endothelial cells. Am J Physiol (1997) 273:H981–988. doi: 10.1152/ajpheart.1997.273.2.H981 9277518

[84] Nourshargh S Hordijk PL Sixt M. Breaching multiple barriers: leukocyte motility through venular walls and the interstitium. Nat Rev Mol Cell Biol (2010) 11:366–78. doi: 10.1038/nrm2889 20414258

[85] Wettschureck N Strilic B Offermanns S. Passing the vascular barrier: endothelial signaling processes controlling extravasation. Physiol Rev (2019) 99:1467–525. doi: 10.1152/physrev.00037.2018 31140373

[86] Reglero-Real N Colom B Bodkin JV Nourshargh S. Endothelial cell junctional adhesion molecules: role and regulation of expression in inflammation. Arterioscler Thromb Vasc Biol (2016) 36:2048–57. doi: 10.1161/ATVBAHA.116.307610 PMC5035539 27515379

[87] Sluiter TJ van Buul JD Huveneers S Quax PHA de Vries MR. Endothelial barrier function and leukocyte transmigration in atherosclerosis. Biomedicines (2021) 9(4):328. doi: 10.3390/biomedicines9040328 33804952 PMC8063931

[88] Dejana E Tournier-Lasserve E Weinstein BM. The control of vascular integrity by endothelial cell junctions: molecular basis and pathological implications. Dev Cell (2009) 16:209–21. doi: 10.1016/j.devcel.2009.01.004 19217423

[89] Carmeliet P. Mechanisms of angiogenesis and arteriogenesis. Nat Med (2000) 6:389–95. doi: 10.1038/74651 10742145

[90] Welti J Loges S Dimmeler S Carmeliet P. Recent molecular discoveries in angiogenesis and antiangiogenic therapies in cancer. J Clin Invest (2013) 123:3190–200. doi: 10.1172/JCI70212 PMC3726176 23908119

[91] Melgar-Lesmes P Edelman ER. Monocyte-endothelial cell interactions in the regulation of vascular sprouting and liver regeneration in mouse. J Hepatol (2015) 63:917–25. doi: 10.1016/j.jhep.2015.05.011 PMC4575901 26022689

[92] Schubert SY Benarroch A Monter-Solans J Edelman ER. Primary monocytes regulate endothelial cell survival through secretion of angiopoietin-1 and activation of endothelial Tie2. Arterioscler Thromb Vasc Biol (2011) 31:870–5. doi: 10.1161/ATVBAHA.110.218255 PMC3104028 21273558

[93] Schubert SY Benarroch A Ostvang J Edelman ER. Regulation of endothelial cell proliferation by primary monocytes. Arterioscler Thromb Vasc Biol (2008) 28:97–104. doi: 10.1161/ATVBAHA.107.157537 17991870

[94] Schubert SY Benarroch A Monter-Solans J Edelman ER. Monocyte activation state regulates monocyte-induced endothelial proliferation through met signaling. Blood (2010) 115:3407–12. doi: 10.1182/blood-2009-02-207340 PMC2858488 20190195

[95] Fantin A Vieira JM Gestri G Denti L Schwarz Q Prykhozhij S. Tissue macrophages act as cellular chaperones for vascular anastomosis downstream of VEGF-mediated endothelial tip cell induction. Blood (2010) 116:829–40. doi: 10.1182/blood-2009-12-257832 PMC2938310 20404134

[96] Sass G Koerber K Bang R Guehring H Tiegs G. Inducible nitric oxide synthase is critical for immune-mediated liver injury in mice. J Clin Invest (2001) 107:439–47. doi: 10.1172/JCI10613 PMC199245 11181643

[97] Eliceiri BP Paul R Schwartzberg PL Hood JD Leng J Cheresh DA. Selective requirement for src kinases during VEGF-induced angiogenesis and vascular permeability. Mol Cell (1999) 4:915–24. doi: 10.1016/S1097-2765(00)80221-X 10635317

[98] Sun Z Li X Massena S Kutschera S Padhan N Gualandi L. VEGFR2 induces c-src signaling and vascular permeability *in vivo via* the adaptor protein TSAd. J Exp Med (2012) 209:1363–77. doi: 10.1084/jem.20111343 PMC3405501 22689825

[99] Thurston G Rudge JS Ioffe E Zhou H Ross L Croll SD. Angiopoietin-1 protects the adult vasculature against plasma leakage. Nat Med (2000) 6:460–3. doi: 10.1038/74725 10742156

[100] Radeva MY Waschke J. Mind the gap: mechanisms regulating the endothelial barrier. Acta Physiol (Oxf) (2018) 222:e12860. doi: 10.1111/apha.12860 28231640

[101] Yuan HT Khankin EV Karumanchi SA Parikh SM. Angiopoietin 2 is a partial agonist/antagonist of Tie2 signaling in the endothelium. Mol Cell Biol (2009) 29:2011–22. doi: 10.1128/MCB.01472-08 PMC2663314 19223473

[102] Pauta M Ribera J Melgar-Lesmes P Casals G Rodriguez-Vita J Reichenbach V. Overexpression of angiopoietin-2 in rats and patients with liver fibrosis. therapeutic consequences of its inhibition. Liver Int (2015) 35:1383–92. doi: 10.1111/liv.12505 24612347

[103] Carmeliet P Jain RK. Molecular mechanisms and clinical applications of angiogenesis. Nature (2011) 473:298–307. doi: 10.1038/nature10144 21593862 PMC4049445

[104] Newby AC. Metalloproteinase expression in monocytes and macrophages and its relationship to atherosclerotic plaque instability. Arterioscler Thromb Vasc Biol (2008) 28:2108–14. doi: 10.1161/ATVBAHA.108.173898 18772495

[105] Quintero-Fabian S Arreola R Becerril-Villanueva E Torres-Romero JC Arana-Argaez V Lara-Riegos J. Role of matrix metalloproteinases in angiogenesis and cancer. Front Oncol (2019) 9:1370. doi: 10.3389/fonc.2019.01370 31921634 PMC6915110

[106] Hildebrand F Hubbard WJ Choudhry MA Frink M Pape HC Kunkel SL. Kupffer cells and their mediators: the culprits in producing distant organ damage after trauma-hemorrhage. Am J Pathol (2006) 169:784–94. doi: 10.2353/ajpath.2006.060010 PMC1698811 16936255

[107] Abraham S Yeo M Montero-Balaguer M Paterson H Dejana E Marshall CJ. VE-cadherin-mediated cell-cell interaction suppresses sprouting *via* signaling to MLC2 phosphorylation. Curr Biol (2009) 19:668–74. doi: 10.1016/j.cub.2009.02.057 19345098

[108] Blumenthal A Ehlers S Lauber J Buer J Lange C Goldmann T. The wingless homolog WNT5A and its receptor frizzled-5 regulate inflammatory responses of human mononuclear cells induced by microbial stimulation. Blood (2006) 108:965–73. doi: 10.1182/blood-2005-12-5046 16601243

[109] Holland J Owens T. Signaling through intercellular adhesion molecule 1 (ICAM-1) in a b cell lymphoma line. the activation of Lyn tyrosine kinase and the mitogen-activated protein kinase pathway. J Biol Chem (1997) 272:9108–12. doi: 10.1074/jbc.272.14.9108 9083038

[110] Wimmer R Cseh B Maier B Scherrer K Baccarini M. Angiogenic sprouting requires the fine tuning of endothelial cell cohesion by the raf-1/Rok-alpha complex. Dev Cell (2012) 22:158–71. doi: 10.1016/j.devcel.2011.11.012 PMC3268451 22209329

[111] Hoefer IE van Royen N Rectenwald JE Deindl E Hua J Jost M. Arteriogenesis proceeds *via* ICAM-1/Mac-1- mediated mechanisms. Circ Res (2004) 94:1179–85. doi: 10.1161/01.RES.0000126922.18222.F0 15059933

[112] Li Z Burns AR Smith CW. Lymphocyte function-associated antigen-1-dependent inhibition of corneal wound healing. Am J Pathol (2006) 169:1590–600. doi: 10.2353/ajpath.2006.060415 PMC1780217 17071583

[113] Peters T Sindrilaru A Hinz B Hinrichs R Menke A Al-Azzeh EA. Wound-healing defect of CD18(-/-) mice due to a decrease in TGF-beta1 and myofibroblast differentiation. EMBO J (2005) 24:3400–10. doi: 10.1038/sj.emboj.7600809 PMC1276170 16148944

[114] Angelini DJ Hyun SW Grigoryev DN Garg P Gong P Singh IS. TNF-alpha increases tyrosine phosphorylation of vascular endothelial cadherin and opens the paracellular pathway through fyn activation in human lung endothelia. Am J Physiol Lung Cell Mol Physiol (2006) 291:L1232–1245. doi: 10.1152/ajplung.00109.2006 16891393

[115] Lai JJ Lai KP Chuang KH Chang P Yu IC Lin WJ. Monocyte/macrophage androgen receptor suppresses cutaneous wound healing in mice by enhancing local TNF-alpha expression. J Clin Invest (2009) 119:3739–51. doi: 10.1172/JCI39335 PMC2786793 19907077

[116] Wu Y Hirschi KK. Tissue-resident macrophage development and function. Front Cell Dev Biol (2020) 8:617879. doi: 10.3389/fcell.2020.617879 33490082 PMC7820365

[117] Davies LC Jenkins SJ Allen JE Taylor PR. Tissue-resident macrophages. Nat Immunol (2013) 14:986–95. doi: 10.1038/ni.2705 PMC4045180 24048120

[118] DeFalco T Bhattacharya I Williams AV Sams DM Capel B. Yolk-sac-derived macrophages regulate fetal testis vascularization and morphogenesis. Proc Natl Acad Sci U.S.A. (2014) 111:E2384–2393. doi: 10.1073/pnas.1400057111 PMC4060703 24912173

[119] Plein A Fantin A Denti L Pollard JW Ruhrberg C. Erythro-myeloid progenitors contribute endothelial cells to blood vessels. Nature (2018) 562:223–8. doi: 10.1038/s41586-018-0552-x PMC6289247 30258231

[120] Wang Y Chang T Wu T Xu W Dou G Wang Y. M2 macrophages promote vasculogenesis during retinal neovascularization by regulating bone marrow-derived cells *via* SDF-1/VEGF. Cell Tissue Res (2020) 380:469–86. doi: 10.1007/s00441-019-03166-9 31989253

[121] Melgar-Lesmes P Balcells M Edelman ER. Implantation of healthy matrix-embedded endothelial cells rescues dysfunctional endothelium and ischaemic tissue in liver engraftment. Gut (2017) 66:1297–305. doi: 10.1136/gutjnl-2015-310409 PMC5288307 26851165

[122] Dalakas E Newsome PN Harrison DJ Plevris JN. Hematopoietic stem cell trafficking in liver injury. FASEB J (2005) 19:1225–31. doi: 10.1096/fj.04-2604rev 16051689

[123] Palis J. Hematopoietic stem cell-independent hematopoiesis: emergence of erythroid, megakaryocyte, and myeloid potential in the mammalian embryo. FEBS Lett (2016) 590:3965–74. doi: 10.1002/1873-3468.12459 27790707

[124] Hoeffel G Ginhoux F. Fetal monocytes and the origins of tissue-resident macrophages. Cell Immunol (2018) 330:5–15. doi: 10.1016/j.cellimm.2018.01.001 29475558

[125] Tober J Koniski A McGrath KE Vemishetti R Emerson R de Mesy-Bentley KK. The megakaryocyte lineage originates from hemangioblast precursors and is an integral component both of primitive and of definitive hematopoiesis. Blood (2007) 109:1433–41. doi: 10.1182/blood-2006-06-031898 PMC1794060 17062726

[126] Takahashi K Yamamura F Naito M. Differentiation, maturation, and proliferation of macrophages in the mouse yolk sac: a light-microscopic, enzyme-cytochemical, immunohistochemical, and ultrastructural study. J Leukoc Biol (1989) 45:87–96. doi: 10.1002/jlb.45.2.87 2536795

[127] Riabov V Gudima A Wang N Mickley A Orekhov A Kzhyshkowska J. Role of tumor associated macrophages in tumor angiogenesis and lymphangiogenesis. Front Physiol (2014) 5:75. doi: 10.3389/fphys.2014.00075 24634660 PMC3942647

[128] Sidibe A Ropraz P Jemelin S Emre Y Poittevin M Pocard M. Angiogenic factor-driven inflammation promotes extravasation of human proangiogenic monocytes to tumours. Nat Commun (2018) 9:355. doi: 10.1038/s41467-017-02610-0 29367702 PMC5783934

[129] Venneri MA De Palma M Ponzoni M Pucci F Scielzo C Zonari E. Identification of proangiogenic TIE2-expressing monocytes (TEMs) in human peripheral blood and cancer. Blood (2007) 109:5276–85. doi: 10.1182/blood-2006-10-053504 17327411

[130] Willenborg S Lucas T van Loo G Knipper JA Krieg T Haase I. CCR2 recruits an inflammatory macrophage subpopulation critical for angiogenesis in tissue repair. Blood (2012) 120:613–25. doi: 10.1182/blood-2012-01-403386 22577176

[131] Shojaei F Wu X Malik AK Zhong C Baldwin ME Schanz S. Tumor refractoriness to anti-VEGF treatment is mediated by CD11b+Gr1+ myeloid cells. Nat Biotechnol (2007) 25:911–20. doi: 10.1038/nbt1323 17664940

[132] Imaizumi T Matsumiya T Fujimoto K Okamoto K Cui X Ohtaki U. Interferon-gamma stimulates the expression of CX3CL1/fractalkine in cultured human endothelial cells. Tohoku J Exp Med (2000) 192:127–39. doi: 10.1620/tjem.192.127 11211312

[133] Ancuta P Wang J Gabuzda D. CD16+ monocytes produce IL-6, CCL2, and matrix metalloproteinase-9 upon interaction with CX3CL1-expressing endothelial cells. J Leukoc Biol (2006) 80:1156–64. doi: 10.1189/jlb.0206125 17056766

[134] Ancuta P Rao R Moses A Mehle A Shaw SK Luscinskas FW. Fractalkine preferentially mediates arrest and migration of CD16+ monocytes. J Exp Med (2003) 197:1701–7. doi: 10.1084/jem.20022156 PMC2193954 12810688

[135] Fraticelli P Sironi M Bianchi G D’Ambrosio D Albanesi C Stoppacciaro A. Fractalkine (CX3CL1) as an amplification circuit of polarized Th1 responses. J Clin Invest (2001) 107:1173–81. doi: 10.1172/JCI11517 PMC209276 11342581

[136] Hanna RN Cekic C Sag D Tacke R Thomas GD Nowyhed H. Patrolling monocytes control tumor metastasis to the lung. Science (2015) 350:985–90. doi: 10.1126/science.aac9407 PMC4869713 26494174

[137] Chouaib S Messai Y Couve S Escudier B Hasmim M Noman MZ. Hypoxia promotes tumor growth in linking angiogenesis to immune escape. Front Immunol (2012) 3:21. doi: 10.3389/fimmu.2012.00021 22566905 PMC3341970

[138] Liao D Johnson RS. Hypoxia: a key regulator of angiogenesis in cancer. Cancer Metastasis Rev (2007) 26:281–90. doi: 10.1007/s10555-007-9066-y 17603752

[139] Yamakawa M Liu LX Date T Belanger AJ Vincent KA Akita GY. Hypoxia-inducible factor-1 mediates activation of cultured vascular endothelial cells by inducing multiple angiogenic factors. Circ Res (2003) 93:664–73. doi: 10.1161/01.RES.0000093984.48643.D7 12958144

[140] Wigerup C Pahlman S Bexell D. Therapeutic targeting of hypoxia and hypoxia-inducible factors in cancer. Pharmacol Ther (2016) 164:152–69. doi: 10.1016/j.pharmthera.2016.04.009 27139518

[141] Welford AF Biziato D Coffelt SB Nucera S Fisher M Pucci F. TIE2-expressing macrophages limit the therapeutic efficacy of the vascular-disrupting agent combretastatin A4 phosphate in mice. J Clin Invest (2011) 121:1969–73. doi: 10.1172/JCI44562 PMC3083764 21490397

[142] Schioppa T Uranchimeg B Saccani A Biswas SK Doni A Rapisarda A. Regulation of the chemokine receptor CXCR4 by hypoxia. J Exp Med (2003) 198:1391–402. doi: 10.1084/jem.20030267 PMC2194248 14597738

[143] Pathria P Louis TL Varner JA. Targeting tumor-associated macrophages in cancer. Trends Immunol (2019) 40:310–27. doi: 10.1016/j.it.2019.02.003 30890304

[144] Petty AJ Yang Y. Tumor-associated macrophages: implications in cancer immunotherapy. Immunotherapy (2017) 9:289–302. doi: 10.2217/imt-2016-0135 28231720 PMC5619052

[145] Murdoch C Muthana M Coffelt SB Lewis CE. The role of myeloid cells in the promotion of tumour angiogenesis. Nat Rev Cancer (2008) 8:618–31. doi: 10.1038/nrc2444 18633355

[146] Gonzalez H Hagerling C Werb Z. Roles of the immune system in cancer: from tumor initiation to metastatic progression. Genes Dev (2018) 32:1267–84. doi: 10.1101/gad.314617.118 PMC6169832 30275043

[147] Noy R Pollard JW. Tumor-associated macrophages: from mechanisms to therapy. Immunity (2014) 41:49–61. doi: 10.1016/j.immuni.2014.06.010 25035953 PMC4137410

[148] Peranzoni E Lemoine J Vimeux L Feuillet V Barrin S Kantari-Mimoun C. Macrophages impede CD8 T cells from reaching tumor cells and limit the efficacy of anti-PD-1 treatment. Proc Natl Acad Sci U.S.A. (2018) 115:E4041–50. doi: 10.1073/pnas.1720948115 PMC5924916 29632196

[149] DeNardo DG Ruffell B. Macrophages as regulators of tumour immunity and immunotherapy. Nat Rev Immunol (2019) 19:369–82. doi: 10.1038/s41577-019-0127-6 PMC7339861 30718830

[150] Coffelt SB Tal AO Scholz A De Palma M Patel S Urbich C. Angiopoietin-2 regulates gene expression in TIE2-expressing monocytes and augments their inherent proangiogenic functions. Cancer Res (2010) 70:5270–80. doi: 10.1158/0008-5472.CAN-10-0012 20530679

[151] Crowther M Brown NJ Bishop ET Lewis CE. Microenvironmental influence on macrophage regulation of angiogenesis in wounds and malignant tumors. J Leukoc Biol (2001) 70:478–90. doi: 10.1189/jlb.70.4.478 11590184

[152] Dirkx AE Oude Egbrink MG Wagstaff J Griffioen AW. Monocyte/macrophage infiltration in tumors: modulators of angiogenesis. J Leukoc Biol (2006) 80:1183–96. doi: 10.1189/jlb.0905495 16997855

[153] Laoui D Van Overmeire E Di Conza G Aldeni C Keirsse J Morias Y. Tumor hypoxia does not drive differentiation of tumor-associated macrophages but rather fine-tunes the M2-like macrophage population. Cancer Res (2014) 74:24–30. doi: 10.1158/0008-5472.CAN-13-1196 24220244

[154] Tannahill GM Curtis AM Adamik J Palsson-McDermott EM McGettrick AF Goel G. Succinate is an inflammatory signal that induces IL-1beta through HIF-1alpha. Nature (2013) 496:238–42. doi: 10.1038/nature11986 PMC4031686 23535595

[155] Vats D Mukundan L Odegaard JI Zhang L Smith KL Morel CR. Oxidative metabolism and PGC-1beta attenuate macrophage-mediated inflammation. Cell Metab (2006) 4:13–24. doi: 10.1016/j.cmet.2006.05.011 16814729 PMC1904486

[156] Wenes M Shang M Di Matteo M Goveia J Martin-Perez R Serneels J. Macrophage metabolism controls tumor blood vessel morphogenesis and metastasis. Cell Metab (2016) 24:701–15. doi: 10.1016/j.cmet.2016.09.008 27773694

[157] Gaidai O Cao Y Loginov S. Global cardiovascular diseases death rate prediction. Curr Probl Cardiol (2023) 48:101622. doi: 10.1016/j.cpcardiol.2023.101622 36724816

[158] Tsao CW Aday AW Almarzooq ZI Anderson CAM Arora P Avery CL. Heart disease and stroke statistics-2023 update: a report from the American heart association. Circulation (2023) 147:e93–e621. doi: 10.1161/CIR.0000000000001123 36695182 PMC12135016

[159] Liu R Shao J. Research progress on risk factors related to intracranial artery, carotid artery, and coronary artery stenosis. Front Cardiovasc Med (2022) 9:970476. doi: 10.3389/fcvm.2022.970476 36386370 PMC9640748

[160] Garcia-Polite F Martorell J Del Rey-Puech P Melgar-Lesmes P O’Brien CC Roquer J. Pulsatility and high shear stress deteriorate barrier phenotype in brain microvascular endothelium. J Cereb Blood Flow Metab (2017) 37:2614–25. doi: 10.1177/0271678X16672482 PMC5531355 27702879

[161] Wilson HM. Macrophages heterogeneity in atherosclerosis - implications for therapy. J Cell Mol Med (2010) 14:2055–65. doi: 10.1111/j.1582-4934.2010.01121.x PMC3822996 20629993

[162] Gerrity RG Naito HK. Ultrastructural identification of monocyte-derived foam cells in fatty streak lesions. Artery (1980) 8:208–14. 7213030

[163] Gupta RM Lee-Kim VS Libby P. The march of monocytes in atherosclerosis: one cell at a time. Circ Res (2020) 126:1324–6. doi: 10.1161/CIRCRESAHA.120.316981 PMC7236482 32379573

[164] Bosch X Jauregui B Villamor N Morales-Ruiz M Ortiz-Perez JT Borras R. Monocyte subsets are differently associated with infarct size, left ventricular function, and the formation of a potentially arrhythmogenic scar in patients with acute myocardial infarction. J Cardiovasc Transl Res (2020) 13:722–30. doi: 10.1007/s12265-019-09944-8 31833003

[165] Swirski FK Robbins CS Nahrendorf M. Development and function of arterial and cardiac macrophages. Trends Immunol (2016) 37:32–40. doi: 10.1016/j.it.2015.11.004 26748179 PMC4706986

[166] Jaiswal S Natarajan P Silver AJ Gibson CJ Bick AG Shvartz E. Clonal hematopoiesis and risk of atherosclerotic cardiovascular disease. N Engl J Med (2017) 377:111–21. doi: 10.1056/NEJMoa1701719 PMC6717509 28636844

[167] Willemsen L de Winther MP. Macrophage subsets in atherosclerosis as defined by single-cell technologies. J Pathol (2020) 250:705–14. doi: 10.1002/path.5392 PMC7217201 32003464

[168] Tabas I Lichtman AH. Monocyte-macrophages and T cells in atherosclerosis. Immunity (2017) 47:621–34. doi: 10.1016/j.immuni.2017.09.008 PMC5747297 29045897

[169] de Gaetano M Crean D Barry M Belton O. M1- and M2-type macrophage responses are predictive of adverse outcomes in human atherosclerosis. Front Immunol (2016) 7:275. doi: 10.3389/fimmu.2016.00275 27486460 PMC4949256

[170] Kuznetsova T Prange KHM Glass CK de Winther MPJ. Transcriptional and epigenetic regulation of macrophages in atherosclerosis. Nat Rev Cardiol (2020) 17:216–28. doi: 10.1038/s41569-019-0265-3 PMC7770754 31578516

[171] Crijns H Vanheule V Proost P. Targeting chemokine-glycosaminoglycan interactions to inhibit inflammation. Front Immunol (2020) 11:483. doi: 10.3389/fimmu.2020.00483 32296423 PMC7138053

[172] Lu X Wang Z Ye D Feng Y Liu M Xu Y. The role of CXC chemokines in cardiovascular diseases. Front Pharmacol (2021) 12:765768. doi: 10.3389/fphar.2021.765768 35668739 PMC9163960

[173] Doring Y van der Vorst EPC Duchene J Jansen Y Gencer S Bidzhekov K. CXCL12 derived from endothelial cells promotes atherosclerosis to drive coronary artery disease. Circulation (2019) 139:1338–40. doi: 10.1161/CIRCULATIONAHA.118.037953 PMC6417827 30865486

[174] Botts SR Fish JE Howe KL. Dysfunctional vascular endothelium as a driver of atherosclerosis: emerging insights into pathogenesis and treatment. Front Pharmacol (2021) 12:787541. doi: 10.3389/fphar.2021.787541 35002720 PMC8727904

[175] Koskinas KC Chatzizisis YS Papafaklis MI Coskun AU Baker AB Jarolim P. Synergistic effect of local endothelial shear stress and systemic hypercholesterolemia on coronary atherosclerotic plaque progression and composition in pigs. Int J Cardiol (2013) 169:394–401. doi: 10.1016/j.ijcard.2013.10.021 24148915 PMC4191915

[176] Franses JW Drosu NC Gibson WJ Chitalia VC Edelman ER. Dysfunctional endothelial cells directly stimulate cancer inflammation and metastasis. Int J Cancer (2013) 133:1334–44. doi: 10.1002/ijc.28146 PMC3707950 23463345

[177] Silvestre-Roig C de Winther MP Weber C Daemen MJ Lutgens E Soehnlein O. Atherosclerotic plaque destabilization: mechanisms, models, and therapeutic strategies. Circ Res (2014) 114:214–26. doi: 10.1161/CIRCRESAHA.114.302355 24385514

[178] Mulligan-Kehoe MJ Simons M. Vasa vasorum in normal and diseased arteries. Circulation (2014) 129:2557–66. doi: 10.1161/CIRCULATIONAHA.113.007189 24934463

[179] Kalluri AS Vellarikkal SK Edelman ER Nguyen L Subramanian A Ellinor PT. Single-cell analysis of the normal mouse aorta reveals functionally distinct endothelial cell populations. Circulation (2019) 140:147–63. doi: 10.1161/CIRCULATIONAHA.118.038362 PMC6693656 31146585

[180] Wu XH He YY Chen ZR He ZY Yan Y He Y. Single-cell analysis of peripheral blood from high-altitude pulmonary hypertension patients identifies a distinct monocyte phenotype. Nat Commun (2023) 14:1820. doi: 10.1038/s41467-023-37527-4 37002243 PMC10066231

[181] Yu YA Malakhau Y Yu CA Phelan SJ Cumming RI Kan MJ. Nonclassical monocytes sense hypoxia, regulate pulmonary vascular remodeling, and promote pulmonary hypertension. J Immunol (2020) 204:1474–85. doi: 10.4049/jimmunol.1900239 PMC7065976 31996456

[182] Florentin J Coppin E Vasamsetti SB Zhao J Tai YY Tang Y. Inflammatory macrophage expansion in pulmonary hypertension depends upon mobilization of blood-borne monocytes. J Immunol (2018) 200:3612–25. doi: 10.4049/jimmunol.1701287 PMC5940510 29632145

[183] Mongirdiene A Liuize A Kasauskas A. Novel knowledge about molecular mechanisms of heparin-induced thrombocytopenia type II and treatment targets. Int J Mol Sci (2023) 24(9):8217. doi: 10.3390/ijms24098217 37175923 PMC10179321

[184] Li Y Yang J Huang Y Ge S Song X Jia R. Cellular heterogeneity and immune microenvironment revealed by single-cell transcriptome in venous malformation and cavernous venous malformation. J Mol Cell Cardiol (2022) 162:130–43. doi: 10.1016/j.yjmcc.2021.09.004 34536440

[185] Xue Y Luo M Hu X Li X Shen J Zhu W. Macrophages regulate vascular smooth muscle cell function during atherosclerosis progression through IL-1beta/STAT3 signaling. Commun Biol (2022) 5:1316. doi: 10.1038/s42003-022-04255-2 36456628 PMC9715630

[186] Balcells M Martorell J Olive C Santacana M Chitalia V Cardoso AA. Smooth muscle cells orchestrate the endothelial cell response to flow and injury. Circulation (2010) 121:2192–9. doi: 10.1161/CIRCULATIONAHA.109.877282 PMC2887340 20458015

[187] Baker AB Ettenson DS Jonas M Nugent MA Iozzo RV Edelman ER. Endothelial cells provide feedback control for vascular remodeling through a mechanosensitive autocrine TGF-beta signaling pathway. Circ Res (2008) 103:289–97. doi: 10.1161/CIRCRESAHA.108.179465 PMC2766078 18583708

[188] Saemisch M Balcells M Riesinger L Nickmann M Bhaloo SI Edelman ER. Subendothelial matrix components influence endothelial cell apoptosis *in vitro* . Am J Physiol Cell Physiol (2019) 316:C210–22. doi: 10.1152/ajpcell.00005.2018 PMC6397340 30566394

[189] Wilcox EC Edelman ER. Substratum interactions modulate interplay between endothelial cell, epithelial cell, and fibroblast phenotype and immunomodulatory function. Biomaterials (2022) 289:121785. doi: 10.1016/j.biomaterials.2022.121785 36099714

[190] Wilcox EC Edelman ER. Substratum interactions determine immune response to allogeneic transplants of endothelial cells. Front Immunol (2022) 13:946794. doi: 10.3389/fimmu.2022.946794 36003373 PMC9393654

[191] Melgar-Lesmes P Garcia-Polite F Del-Rey-Puech P Rosas E Dreyfuss JL Montell E. Treatment with chondroitin sulfate to modulate inflammation and atherogenesis in obesity. Atherosclerosis (2016) 245:82–7. doi: 10.1016/j.atherosclerosis.2015.12.016 PMC4738029 26714044

[192] du Souich P Garcia AG Verges J Montell E. Immunomodulatory and anti-inflammatory effects of chondroitin sulphate. J Cell Mol Med (2009) 13:1451–63. doi: 10.1111/j.1582-4934.2009.00826.x PMC3828858 19522843

[193] Melgar-Lesmes P Sanchez-Herrero A Lozano-Juan F de la Torre Hernandez JM Montell E Jimenez W. Chondroitin sulphate attenuates atherosclerosis in ApoE knockout mice involving cellular regulation of the inflammatory response. Thromb Haemost (2018) 118:1329–39. doi: 10.1055/s-0038-1657753 PMC6238215 29874688

[194] Lopez-Moya M Melgar-Lesmes P Kolandaivelu K de la Torre Hernandez JM Edelman ER Balcells M. Optimizing glutaraldehyde-fixed tissue heart valves with chondroitin sulfate hydrogel for endothelialization and shielding against deterioration. Biomacromolecules (2018) 19:1234–44. doi: 10.1021/acs.biomac.8b00077 PMC6198652 29539266

[195] Conway C Desany GJ Bailey LR Keating JH Baker BL Edelman ER. Fracture in drug-eluting stents increases focal intimal hyperplasia in the atherosclerosed rabbit iliac artery. Catheter Cardiovasc Interv (2019) 93:278–85. doi: 10.1002/ccd.27726 PMC6363887 30244502

[196] Tzafriri AR Garcia-Polite F Li X Keating J Balaguer JM Zani B. Defining drug and target protein distributions after stent-based drug release: durable versus deployable coatings. J Control Release (2018) 274:102–8. doi: 10.1016/j.jconrel.2018.02.007 PMC5847480 29421608

[197] Wagner N Wagner KD. Pharmacological utility of PPAR modulation for angiogenesis in cardiovascular disease. Int J Mol Sci (2023) 24(3):2345. doi: 10.3390/ijms24032345 36768666 PMC9916802

[198] He H Wang J Yannie PJ Korzun WJ Yang H Ghosh S. Nanoparticle-based “Two-pronged” approach to regress atherosclerosis by simultaneous modulation of cholesterol influx and efflux. Biomaterials (2020) 260:120333. doi: 10.1016/j.biomaterials.2020.120333 32853832 PMC7530139

[199] He H Yuan Q Bie J Wallace RL Yannie PJ Wang J. Development of mannose functionalized dendrimeric nanoparticles for targeted delivery to macrophages: use of this platform to modulate atherosclerosis. Transl Res (2018) 193:13–30. doi: 10.1016/j.trsl.2017.10.008 29172034 PMC6198660

[200] Leuschner F Dutta P Gorbatov R Novobrantseva TI Donahoe JS Courties G. Therapeutic siRNA silencing in inflammatory monocytes in mice. Nat Biotechnol (2011) 29:1005–10. doi: 10.1038/nbt.1989 PMC3212614 21983520

[201] Wu Z Chen C Luo J Davis JRJ Zhang B Tang L. EGFP-EGF1-conjugated poly (lactic-co-glycolic acid) nanoparticles as a carrier for the delivery of CCR2- shRNA to atherosclerotic macrophage *in vitro* . Sci Rep (2020) 10:19636. doi: 10.1038/s41598-020-76416-4 33184330 PMC7661524

[202] Tao W Yurdagul A Jr. Kong N Li W Wang X Doran AC. siRNA nanoparticles targeting CaMKIIgamma in lesional macrophages improve atherosclerotic plaque stability in mice. Sci Transl Med (2020) 12(553):eaay1063. doi: 10.1126/scitranslmed.aay1063 32718990 PMC7476570

[203] Kurniawan DW Jajoriya AK Dhawan G Mishra D Argemi J Bataller R. Therapeutic inhibition of spleen tyrosine kinase in inflammatory macrophages using PLGA nanoparticles for the treatment of non-alcoholic steatohepatitis. J Control Release (2018) 288:227–38. doi: 10.1016/j.jconrel.2018.09.004 30219279

[204] Bartneck M Scheyda KM Warzecha KT Rizzo LY Hittatiya K Luedde T. Fluorescent cell-traceable dexamethasone-loaded liposomes for the treatment of inflammatory liver diseases. Biomaterials (2015) 37:367–82. doi: 10.1016/j.biomaterials.2014.10.030 25453965

[205] Moreno-Lanceta A Medrano-Bosch M Simon-Codina B Barber-Gonzalez M Jimenez W Melgar-Lesmes P. PPAR-gamma agonist GW1929 targeted to macrophages with dendrimer-graphene nanostars reduces liver fibrosis and inflammation. Pharmaceutics (2023) 15(5):1452. doi: 10.3390/pharmaceutics15051452 37242695 PMC10223394

[206] Wang J Pan W Wang Y Lei W Feng B Du C. Enhanced efficacy of curcumin with phosphatidylserine-decorated nanoparticles in the treatment of hepatic fibrosis. Drug Delivery (2018) 25:1–11. doi: 10.1080/10717544.2017.1399301 29214887 PMC6058669

[207] Melgar-Lesmes P Luquero A Parra-Robert M Mora A Ribera J Edelman ER. Graphene-dendrimer nanostars for targeted macrophage overexpression of metalloproteinase 9 and hepatic fibrosis precision therapy. Nano Lett (2018) 18:5839–45. doi: 10.1021/acs.nanolett.8b02498 PMC6377857 30096241

[208] Gao DY Lin Ts T Sung YC Liu YC Chiang WH Chang CC. CXCR4-targeted lipid-coated PLGA nanoparticles deliver sorafenib and overcome acquired drug resistance in liver cancer. Biomaterials (2015) 67:194–203. doi: 10.1016/j.biomaterials.2015.07.035 26218745

[209] Wang Y Tiruthani K Li S Hu M Zhong G Tang Y. mRNA delivery of a bispecific single-domain antibody to polarize tumor-associated macrophages and synergize immunotherapy against liver malignancies. Adv Mater (2021) 33:e2007603. doi: 10.1002/adma.202007603 33945178 PMC8240965

[210] Chen W Schilperoort M Cao Y Shi J Tabas I Tao W. Macrophage-targeted nanomedicine for the diagnosis and treatment of atherosclerosis. Nat Rev Cardiol (2022) 19:228–49. doi: 10.1038/s41569-021-00629-x PMC8580169 34759324

[211] Joseph SB Castrillo A Laffitte BA Mangelsdorf DJ Tontonoz P. Reciprocal regulation of inflammation and lipid metabolism by liver X receptors. Nat Med (2003) 9:213–9. doi: 10.1038/nm820 12524534

[212] Jarr KU Ye J Kojima Y Ye Z Gao H Schmid S. The pleiotropic benefits of statins include the ability to reduce CD47 and amplify the effect of pro-efferocytic therapies in atherosclerosis. Nat Cardiovasc Res (2022) 1:253–62. doi: 10.1038/s44161-022-00023-x PMC9390974 35990913

[213] Zang X Cheng M Zhang X Chen X. Targeting macrophages using nanoparticles: a potential therapeutic strategy for atherosclerosis. J Mater Chem B (2021) 9:3284–94. doi: 10.1039/D0TB02956D 33881414

[214] Gines P Krag A Abraldes JG Sola E Fabrellas N Kamath PS. Liver cirrhosis. Lancet (2021) 398:1359–76. doi: 10.1016/S0140-6736(21)01374-X 34543610

[215] Fernandez-Varo G Ros J Morales-Ruiz M Cejudo-Martin P Arroyo V Sole M. Nitric oxide synthase 3-dependent vascular remodeling and circulatory dysfunction in cirrhosis. Am J Pathol (2003) 162:1985–93. doi: 10.1016/S0002-9440(10)64331-3 PMC1868141 12759254

[216] Principe A Melgar-Lesmes P Fernandez-Varo G del Arbol LR Ros J Morales-Ruiz M. The hepatic apelin system: a new therapeutic target for liver disease. Hepatology (2008) 48:1193–201. doi: 10.1002/hep.22467 18816630

[217] Balasch J Guimera M Martinez-Pasarell O Ros J Vanrell JA Jimenez W. Adrenomedullin and vascular endothelial growth factor production by follicular fluid macrophages and granulosa cells. Hum Reprod (2004) 19:808–14. doi: 10.1093/humrep/deh204 15016771

[218] Jimenez W Ros J Morales-Ruiz M Navasa M Sole M Colmenero J. Nitric oxide production and inducible nitric oxide synthase expression in peritoneal macrophages of cirrhotic patients. Hepatology (1999) 30:670–6. doi: 10.1002/hep.510300310 10462373

[219] Cejudo-Martin P Morales-Ruiz M Ros J Navasa M Fernandez-Varo G Fuster J. Hypoxia is an inducer of vasodilator agents in peritoneal macrophages of cirrhotic patients. Hepatology (2002) 36:1172–9. doi: 10.1053/jhep.2002.36371 12395327

[220] Cejudo-Martin P Ros J Navasa M Fernandez J Fernandez-Varo G Ruiz-del-Arbol L. Increased production of vascular endothelial growth factor in peritoneal macrophages of cirrhotic patients with spontaneous bacterial peritonitis. Hepatology (2001) 34:487–93. doi: 10.1053/jhep.2001.27093 11526533

[221] Reichenbach V Munoz-Luque J Ros J Casals G Navasa M Fernandez-Varo G. Bacterial lipopolyshaccaride inhibits CB2 receptor expression in human monocytic cells. Gut (2013) 62:1089–91. doi: 10.1136/gutjnl-2012-303662 23348962

[222] Reichenbach V Ros J Fernandez-Varo G Casals G Melgar-Lesmes P Campos T. Prevention of fibrosis progression in CCl4-treated rats: role of the hepatic endocannabinoid and apelin systems. J Pharmacol Exp Ther (2012) 340:629–37. doi: 10.1124/jpet.111.188078 PMC11047215 22160265

[223] Melgar-Lesmes P Perramon M Jimenez W. Roles of the hepatic endocannabinoid and apelin systems in the pathogenesis of liver fibrosis. Cells (2019) 8(11):1311. doi: 10.3390/cells8111311 31653030 PMC6912778

[224] Ju C Tacke F. Hepatic macrophages in homeostasis and liver diseases: from pathogenesis to novel therapeutic strategies. Cell Mol Immunol (2016) 13:316–27. doi: 10.1038/cmi.2015.104 PMC4856798 26908374

[225] Huang R Zhang X Gracia-Sancho J Xie WF. Liver regeneration: cellular origin and molecular mechanisms. Liver Int (2022) 42:1486–95. doi: 10.1111/liv.15174 35107210

[226] Diehl AM. Neighborhood watch orchestrates liver regeneration. Nat Med (2012) 18:497–9. doi: 10.1038/nm.2719 22481408

[227] Thompson MD Monga SP. WNT/beta-catenin signaling in liver health and disease. Hepatology (2007) 45:1298–305. doi: 10.1002/hep.21651 17464972

[228] Yang J Mowry LE Nejak-Bowen KN Okabe H Diegel CR Lang RA. Beta-catenin signaling in murine liver zonation and regeneration: a wnt-wnt situation! Hepatology (2014) 60:964–76. doi: 10.1002/hep.27082 PMC4139486 24700412

[229] Zhang W Zhu X Tang Y Li J Miao C Gong J. Kupffer cells depletion alters cytokine expression and delays liver regeneration after radio-frequency-assisted liver partition with portal vein ligation. Mol Immunol (2022) 144:71–7. doi: 10.1016/j.molimm.2022.02.016 35203023

[230] Matsuo T Yamaguchi S Mitsui S Emi A Shimoda F Okamura H. Control mechanism of the circadian clock for timing of cell division in vivo. Science (2003) 302:255–9. doi: 10.1126/science.1086271 12934012

[231] Bentley K Franco CA Philippides A Blanco R Dierkes M Gebala V. The role of differential VE-cadherin dynamics in cell rearrangement during angiogenesis. Nat Cell Biol (2014) 16:309–21. doi: 10.1038/ncb2926 24658686

[232] Duffield JS Forbes SJ Constandinou CM Clay S Partolina M Vuthoori S. Selective depletion of macrophages reveals distinct, opposing roles during liver injury and repair. J Clin Invest (2005) 115:56–65. doi: 10.1172/JCI200522675 15630444 PMC539199

[233] Ramachandran P Iredale JP Fallowfield JA. Resolution of liver fibrosis: basic mechanisms and clinical relevance. Semin Liver Dis (2015) 35:119–31. doi: 10.1055/s-0035-1550057 25974898

[234] Ramachandran P Dobie R Wilson-Kanamori JR Dora EF Henderson BEP Luu NT. Resolving the fibrotic niche of human liver cirrhosis at single-cell level. Nature (2019) 575:512–8. doi: 10.1038/s41586-019-1631-3 PMC6876711 31597160

[235] Bray F Ferlay J Soerjomataram I Siegel RL Torre LA Jemal A. Global cancer statistics 2018: GLOBOCAN estimates of incidence and mortality worldwide for 36 cancers in 185 countries. CA Cancer J Clin (2018) 68:394–424. doi: 10.3322/caac.21492 30207593

[236] Engblom C Pfirschke C Pittet MJ. The role of myeloid cells in cancer therapies. Nat Rev Cancer (2016) 16:447–62. doi: 10.1038/nrc.2016.54 27339708

[237] Zheng H Peng X Yang S Li X Huang M Wei S. Targeting tumor-associated macrophages in hepatocellular carcinoma: biology, strategy, and immunotherapy. Cell Death Discovery (2023) 9:65. doi: 10.1038/s41420-023-01356-7 36792608 PMC9931715

[238] Zhang Q He Y Luo N Patel SJ Han Y Gao R. Landscape and dynamics of single immune cells in hepatocellular carcinoma. Cell (2019) 179:829–845 e820. doi: 10.1016/j.cell.2019.10.003 31675496

[239] van der Heide D Weiskirchen R Bansal R. Therapeutic targeting of hepatic macrophages for the treatment of liver diseases. Front Immunol (2019) 10:2852. doi: 10.3389/fimmu.2019.02852 31849997 PMC6901832

[240] Huang Y Wang T Yang J Wu X Fan W Chen J. Current strategies for the treatment of hepatocellular carcinoma by modulating the tumor microenvironment *via* nano-delivery systems: a review. Int J Nanomed (2022) 17:2335–52. doi: 10.2147/IJN.S363456 PMC9128750 35619893

